# APEX2-based proximity proteomic analysis identifies candidate interactors for *Plasmodium falciparum* knob-associated histidine-rich protein in infected erythrocytes

**DOI:** 10.1038/s41598-024-61295-w

**Published:** 2024-05-16

**Authors:** Sébastien Charneau, Lucas Silva de Oliveira, Zenon Zenonos, Christine S. Hopp, Izabela M. D. Bastos, Damarys Loew, Bérangère Lombard, Ariane Pandolfo Silveira, Giovanna de Carvalho Nardeli Basílio Lobo, Sônia Nair Bao, Philippe Grellier, Julian C. Rayner

**Affiliations:** 1 Laboratory of Biochemistry and Protein Chemistry, Department of Cell Biology, Institute of Biology, University of Brasília, Brasília, 70910-900 Brazil; 2 UMR 7245 MCAM Molecules of Communication and Adaptation of Microorganisms, Muséum National d’Histoire Naturelle, CNRS, 75231 Paris Cedex 05, France; 3 Wellcome Sanger Institute, Wellcome Genome Campus, Hinxton, Cambridgeshire, CB10 1SA UK; 4 Laboratory of Host Pathogen Interaction, Department of Cell Biology, Institute of Biology, University of Brasília, Brasília, 70910-900 Brazil; 5 Institut Curie, Centre de Recherche, PSL Research University, CurieCoreTech Mass Spectrometry Proteomics, 26 rue d’Ulm, 75248 Paris Cedex 05, France; 6 Laboratory of Microscopy and Microanalysis, Department of Cell Biology, Institute of Biology, University of Brasilia, Brasília, 70910-900 Brazil; 7 Cambridge Institute for Medical Research, University of Cambridge, Hills Road, Cambridge, CB2 0XY UK; 8 Present Address: Biologics Engineering, Oncology R&D, AstraZenecaGranta Park, Cambridge, UK; 9 Present Address: Protozoa Immunology, Bernhard Nocht Institute for Tropical Medicine, Hamburg, Germany

**Keywords:** KAHRP, Protein–protein interactions, APEX2, CRISPR-Cas9, Exported proteins, Parasite biology, Protein-protein interaction networks, CRISPR-Cas9 genome editing

## Abstract

The interaction of *Plasmodium falciparum—*infected red blood cells (iRBCs) with the vascular endothelium plays a crucial role in malaria pathology and disease. KAHRP is an exported *P. falciparum* protein involved in iRBC remodelling, which is essential for the formation of protrusions or “knobs” on the iRBC surface. These knobs and the proteins that are concentrated within them allow the parasites to escape the immune response and host spleen clearance by mediating cytoadherence of the iRBC to the endothelial wall, but this also slows down blood circulation, leading in some cases to severe cerebral and placental complications. In this work, we have applied genetic and biochemical tools to identify proteins that interact with *P. falciparum* KAHRP using enhanced ascorbate peroxidase 2 (APEX2) proximity-dependent biotinylation and label-free shotgun proteomics. A total of 30 potential KAHRP-interacting candidates were identified, based on the assigned fragmented biotinylated ions. Several identified proteins have been previously reported to be part of the Maurer’s clefts and knobs, where KAHRP resides. This study may contribute to a broader understanding of *P. falciparum* protein trafficking and knob architecture and shows for the first time the feasibility of using APEX2-proximity labelling in iRBCs.

## Introduction

Malaria remains a significant global health problem, and the most severe parasite associated disease. Worldwide, 247 million malaria cases and 619,000 deaths were estimated in 2021 due to malaria, mainly caused by *Plasmodium falciparum*^1^. In the last decades, many efforts to develop vaccines against malaria have been employed^2–4^, but until a highly efficacious vaccine has been developed, active drug treatment to cure malaria infections remains a central part of malaria control^5,6^. However, historically several antimalarials have been lost to clinical use by the repeated emergence and spread of drug resistance, and the current frontline antimalarial, artemisinin, is now also under threat^1^. A better understanding of malaria biology and pathogenesis is needed to guide the search for new drugs and vaccines.

In *P. falciparum* infected red blood cells (iRBCs), parasite-encoded adhesion ligands are localised to protrusions, so-called knobs, on the iRBC surface, which lead to the formation of rosettes (an aggregation of iRBCs and/or uninfected RBCs)^7^ as well as the cytoadherence of iRBCs to endothelial cells, allowing the parasite to avoid spleen clearance mechanisms, and causing obstruction in the microvasculature^8,9^. These ligands are also highly variable, driven by a complex process of antigenic variation which allows iRBCs to evade the adaptive immune response^10^. The formation of knobs is only one aspect of iRBC remodelling, which involves the export of hundreds of *P. falciparum* proteins to the iRBC^11,12^, primarily through tubulovesicular network vesicles termed Maurer’s clefts (MC)^8,13^.

KAHRP (*knob-associated histidine rich protein*) is widely thought to be the backbone of the cytoadherence machinery^14–16^. Knobs are organized in a spiral-shape ring structure comprising modular KAHRP units that is assembled as the parasite matures, with up to 60 KAHRP units per knob^17^. KAHRP is a non-essential gene for parasite growth in vitro, but plays a critical role in pathogenesis in vivo^18^. How KAHRP catalyses the formation of knobs is still not completely understood. It is presumed to interact with additional *P. falciparum* exported proteins to do so, but a complete map of KAHRP-interacting proteins has not yet been established.

In recent years, proximity-dependent biotinylation techniques have increasingly used to identify protein–protein interactions. One such approach, BioID, uses a promiscuous biotin ligase fused to a protein of interest expressed in living cells, where it forms an amide bond between reactive biotin and lysine residues (K) of proximal endogenous proteins. Biotinylated proteins are then captured by biotin-affinity, and mass-spectrometry based proteomics is used to identify neighbouring and potentially interacting proteins of the BioID tagged targets^19,20^. This technology has been showing its usefulness in the study of Apicomplexan parasites, including *P. falciparum*^21–23^, *Plasmodium berghei*^24^ and *Toxoplasma gondii*^25,26^. For instance, BioID has been used to define the proteomes of the apicoplast, parasitophorous vacuole and rhoptries in *P. falciparum*^21,22,27^.

An alternative proximity-dependent biotinylation technique is based on the use of the engineered ascorbate-peroxidase (APEX2)^28–30^. The main difference between BioID and APEX2 lie in the incubation time for the biotinylation reaction. While BioID has a labelling period of 15–18 h, APEX2 has a shorter labelling period of the biotin phenoxyl radical of 1 min and, a tagging radius smaller than 20 nm^20,28^. APEX2 is ideal to study short-lived events, however, the labelling appear to be more difficult compared to BioID^20^.

In this study, we aimed to implement APEX2 proximity tagging followed by label-free mass-spectrometry based proteomics to identify candidate interactors of KAHRP, in order to expand understanding of potential KAHRP interactors. We modified the endogenous *kahrp* gene using CRISPR/Cas9 genome engineering to insert both a C-terminal APEX2 domain and FLAG tag, to allow us to follow its location by immunofluorescence. Carrying out in vivo biotinylation in this strain followed by mass spectrometry to specifically identify only biotinylated peptides, we classified 30 proteins as potential interacting partners for KAHRP. Some of these proteins have previously been shown to localize at MC by immunofluorescence or proteomic analyses, but its evidence as a KAHRP interactor was never investigated. These interactions may occur either in the knobs, or during KAHRP transport to its final subcellular location*.* In summary, we demonstrated for the first time the feasibility of using APEX2-proximity labelling in *P. falciparum*.

## Results

### Construction and validation of KAHRP-FLAG-APEX2 expressing parasites

**Figure 1:**
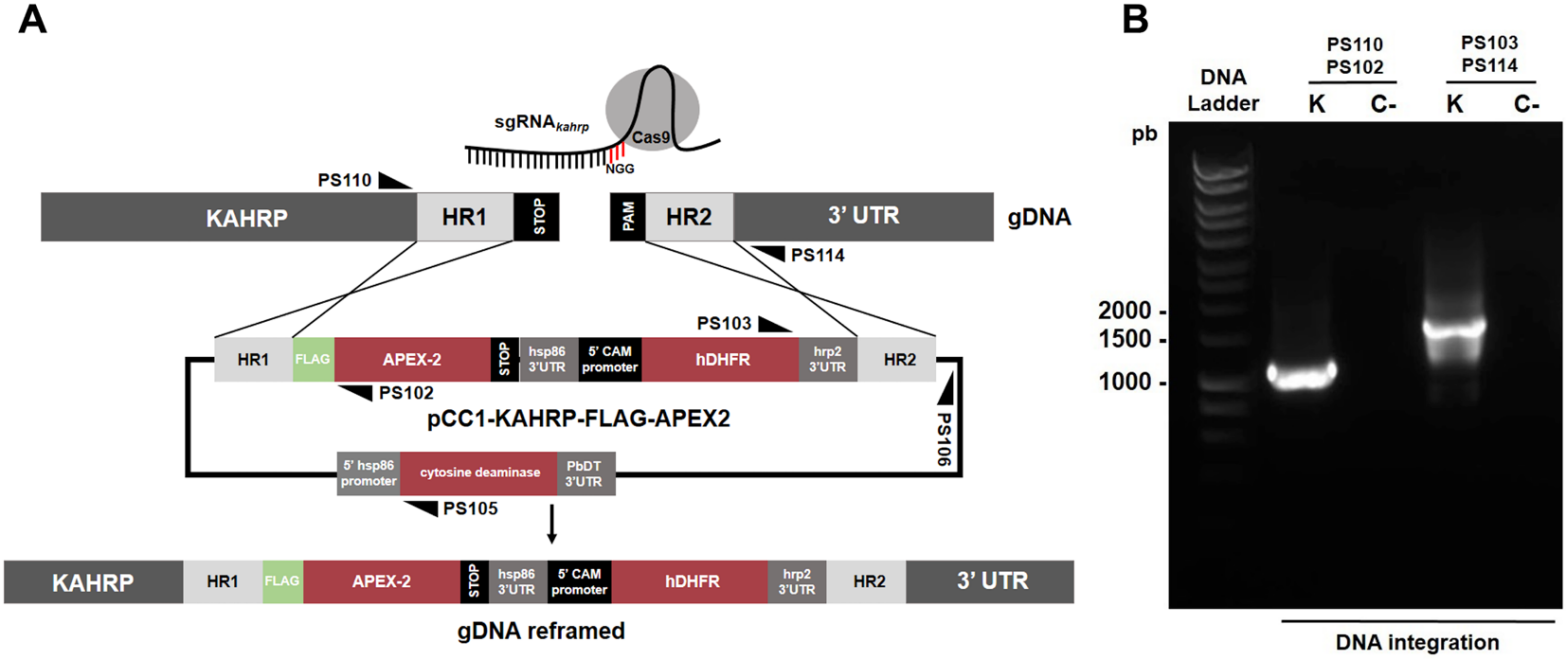
CRISPR/Cas9 guided knock-in scheme to tag the *Plasmodium falciparum kahrp* gene with APEX2. (**A**) A schematic figure illustrating integration of the cassette from the pCC1-KAHRP-FLAG-APEX2 vector into parasite gDNA, resulting in integration of the FLAG tag and APEX2 domain at the 3′ end of the gene, in frame with the KAHRP ORF. (**B**) PCR validation of cassette integration. Legend: K (KAHRP-FLAG-APEX2) and C- (negative control).

The plasmids pCC1-KAHRP-FLAG-APEX2 and pDC2-U6-Cas9-gRNA_*kahrp*_ were co-transfected into the *P. falciparum* 3D7 strain to generate a transgenic strain in which FLAG and APEX2 tags were inserted at the 3′ end of the endogenous *kahrp* gene, creating a chimeric KAHRP-FLAG-APEX2 gene (Fig. 1A). The first transgenic parasites were observed after 21 days under drug pressure. Diagnostic PCR confirmed integration of the cassette to the endogenous KAHRP locus—amplicons from the primers PS110/PS102 and PS103/PS114 of 1.1 kb and 1.6 kb, respectively, confirming correct integration (Fig. 1B).

To test the ability of the integrated APEX2 enzyme to catalyse biotinylation, enriched late-stage KAHRP-FLAG-APEX2 parasites were incubated for 30 min in the presence or absence of Biotin-Phenol (BP+ /−), followed by 1 min of oxidising in the presence or absence of H_2_O_2_. Samples were therefore created from four conditions: BP−/H_2_O_2_− (both without BP and H_2_O_2_); BP−/H_2_O_2_+ (without BP and in the presence of H_2_O_2_); BP+/H_2_O_2_− (in the presence of BP and without H_2_O_2_); and BP+/H_2_O_2_+ (both in the presence of BP and H_2_O_2_)—biotinylation is only expected in the presence of both BP and H_2_O_2_. After quenching, iRBCs were solubilised using saponin treatment, which disrupts the RBC membrane but not the parasite membrane, allowing release of the RBC cytosol with its high levels of haemoglobin. The saponin-treated pellet, which contains both parasites and membrane fragments from the iRBC such as Maurer’s Clefts, was then solubilised using RIPA buffer (RIPA-soluble proteins) and separated by NuPAGE.

**Figure 2:**
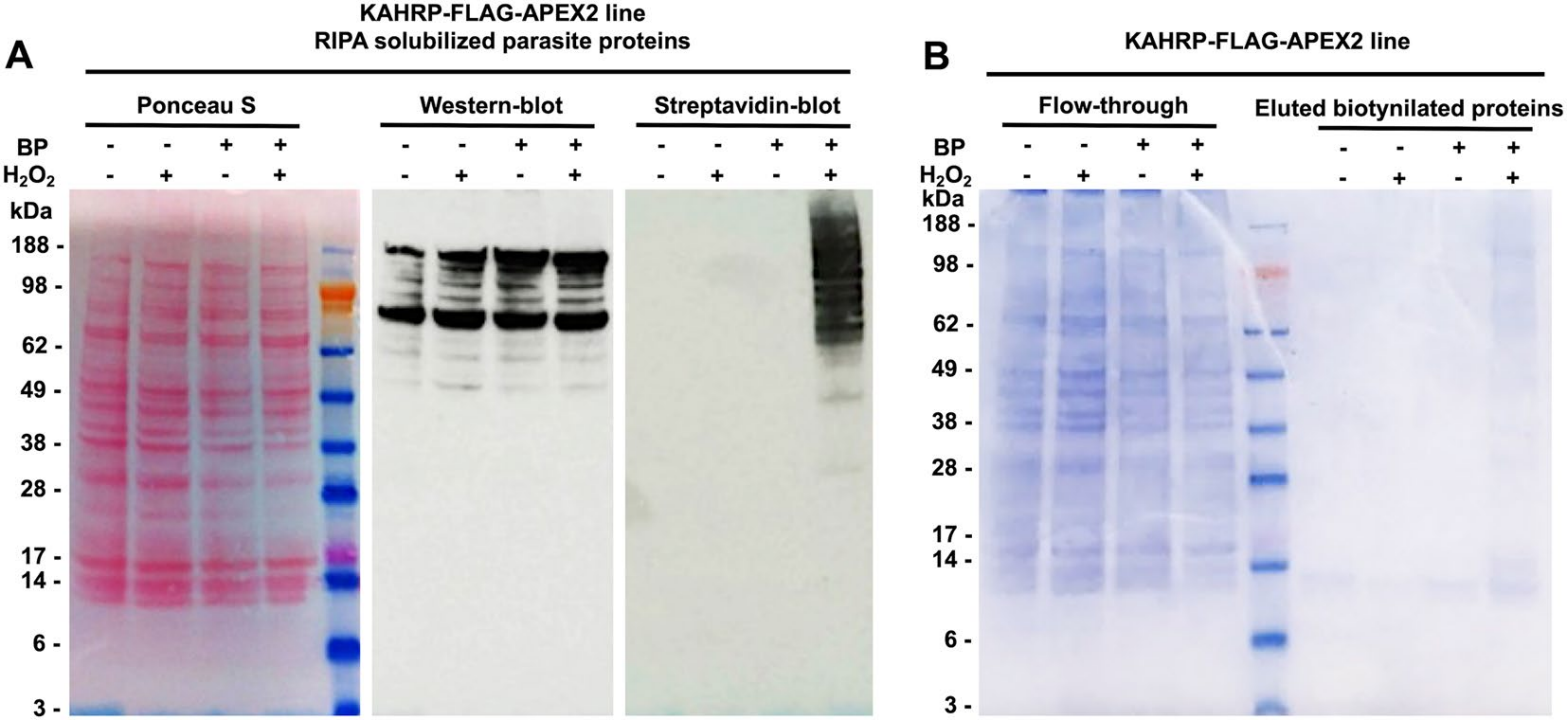
Confirmation of chimeric protein expression and biotinylation activity. (**A**) Ponceau S stained nitrocellulose membrane (left panel), Western-blot (middle panel, anti-FLAG-HRP dilution at 1:800) and streptavidin-blot (right panel, streptavidin-HRP dilution at 1:10,000) of KAHRP-FLAG-APEX2 parasites treated and/or not treated with BP and H_2_O_2_. In all cases, material was generated by saponin lysis of iRBCs, which releases RBC cytosolic contents but retains parasite and exported membrane material, followed by solubilisation of all retained material using RIPA buffer. (**B**) NuPAGE of material enriched from the KAHRP-FLAG-APEX2 line following biotinylation, lysis with saponin and then RIPA, and purification of biotinylated proteins using streptavidin-coated beads enrichment. Eluted proteins stained by Coomassie-blue were only detected in the material from the BP+/H_2_O_2_+ condition (right panel). Legend for all gels/blots: first lane BP−/H_2_O_2_− (not treated with BP or H_2_O_2_); second lane BP−/H_2_O_2_+ (not treated with BP, treated with H_2_O_2_); third lane BP+/H_2_O_2_− (treated with BP but not treated with H_2_O_2_); fourth lane BP+/H_2_O_2_+ (treated with both BP and H_2_O_2_).

Anti-FLAG tag antibodies confirmed the expression of the chimeric protein KAHRP-FLAG-APEX2 in all conditions (Fig. 2A), although the chimeric protein ran higher than predicted molecular mass of 100 kDa, most likely due to charged residues in the C-terminus of KAHRP, or the histidine-sequence repeats, as similar repeats are well-known to produce atypical migration of other proteins in *P. falciparum*^31^. Multiple bands of smaller size were also detected, suggesting partial proteolytic cleavage. It is not known whether this occurred within the parasite or post-lysis, although it should be noted that proteolytic processing is a common feature in *P. falciparum* proteins. Wildtype *P. falciparum* 3D7 strain parasites and a *P. falciparum* transfectant strain in which FLAG-APEX2 was expressed from an episome (Fig. S1A), unlinked to any endogenous protein, were used as controls and show clearly different sized products in Western blot (Fig. S1B), confirming the bands in Fig. 2A are specific to the presence of KAHRP-FLAG-APEX2.

To test the efficacy of the APEX2 biotinylation assay, RIPA-soluble proteins were probed with streptavidin-HRP to label biotinylated proteins. Only the condition in which both the BP substrate and the oxidizing agent H_2_O_2_ were present revealed biotinylated proteins, with multiple bands over a large molecular weight range, suggesting biotinylation of multiple proteins (Fig. 2A). In the episome-based FLAG-APEX2 line, biotinylated proteins were also observed by streptavidin-HRP blot only in the presence of BP and H_2_O_2_ (Fig. S1B). No signal was observed in the negative controls, in which either BP or H_2_O_2_ were omitted in the KAHRP-FLAG-APEX2 line (Fig. 2A) or in wildtype 3D7 parasites (Fig. S1B). Biotinylated proteins were enriched using streptavidin-coated beads from all KAHRP-FLAG-APEX2 samples from all four conditions—again, protein material were observed only in the lane containing the sample treated with both BP and H_2_O_2_ (Fig. 2B). This establishes that APEX2-tagged KAHRP catalyses biotinylation of proteins within the parasite, and that this biotinylation is dependent on incubation with H_2_O_2_ and the BP substrate.

### Localisation of KAHRP-FLAG-APEX2 and biotinylated proteins by confocal immunofluorescence

**Figure 3:**
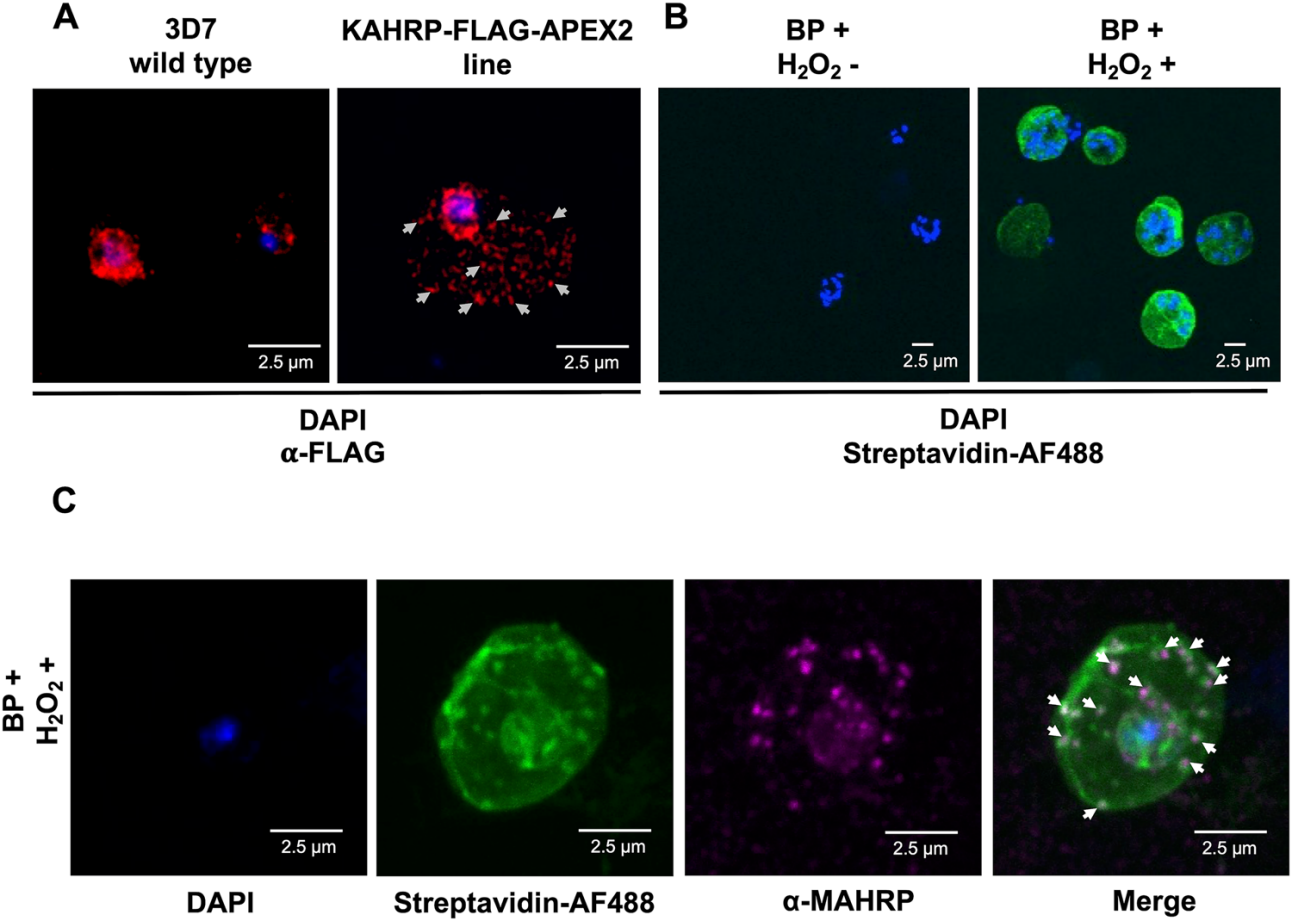
IFA of KAHRP-FLAG-APEX2 and biotinylated candidate interactors of KAHRP. (**A**) 3D7 wild type and KAHRP-FLAG-APEX2 lines incubated with DAPI and anti-FLAG antibodies, followed by detection with Alexa Fluor 555-conjugated anti-mouse antibody. KAHRP-FLAG-APEX2 chimeric protein displayed punctuate localization throughout the parasitized erythrocyte (grey arrows as examples). (**B**) BP+/H_2_O_2_− and BP+/H_2_O_2_+ treated KAHRP-FLAG-APEX2 parasites were incubated with DAPI and streptavidin-AF488. As expected, fluorescence, indicating the presence of biotinylated proteins, was observed only in the BP+/H_2_O_2_+ treated parasites. (**C**) BP+/H_2_O_2_+ treated KAHRP-FLAG-APEX2 parasites were stained with DAPI, streptavidin-AF488 and anti-MAHRP antibody followed by detection with Alexa-Fluor 546-conjugated anti-rabbit IgG donkey antibody. A clear co-localization between biotinylated proteins (green) and MAHRP (purple) was observed, suggesting that KAHRP interacting partners and MAHRP share the same vesicular compartment in the Maurer’s clefts (merge, white arrows as examples).

Immunofluorescence assays (IFA) using anti-FLAG antibodies showed a punctate distribution of KAHRP-FLAG-APEX2 inside the iRBC cytoplasm (Fig. 3A, grey arrows), consistent with previous reports of KAHRP location^32^. Staining was also visible inside the parasite near the nucleus, potentially representing KAHRP that was being synthesised, however, similar staining was also present with anti-FLAG antibodies in wild-type parasites, suggesting the labelling is non-specific (Fig. 3A). By contrast the punctate distribution of anti-FLAG staining was absent from 3D7 parasites, suggesting that this localisation inside the iRBC cytoplasm is specific to the expression of KAHRP-FLAG-APEX2.

To establish the localisation of proteins biotinylated by KAHRP-FLAG-APEX2, we used IFA and fluorescently-tagged streptavidin (streptavidin AF488) to detect proteins that were labelled after incubation with H_2_O_2_ and the BP substrate. Structures within the iRBC cytoplasm were present in schizonts treated with BP+/H_2_O_2_+, but not schizonts from which H_2_O_2_ was omitted, confirming that the labelling was dependent on APEX2 activity (Fig. 3B). Co-localisation of streptavidin-AF488 with antibodies against membrane-associated histidine-rich protein (MAHRP), a specific marker of MC known to colocalize with KAHRP^33–35^, showed that proteins biotinylated by KAHRP-FLAG-APEX2 were partially co-localised to the MC (Fig. 3C, white arrows). Conversely, IFAs using streptavidin-AF488 with the parasite line expressing episome-based FLAG-APEX2 showed biotinylated proteins were primarily localised to the cytosol, and did not co-localise with anti-MAHRP (Fig. S2). In this parasite line, a partial co-localization between biotinylated proteins and the Golgi apparatus at the perinuclear regions of *P. falciparum* segmented schizonts was observed using an antibody raised against the ERD2^36–39^ (Fig. S2).

### KAHRP-FLAG-APEX2 parasites produce iRBCs that display knobs and bind to human endothelial cells

**Figure 4:**
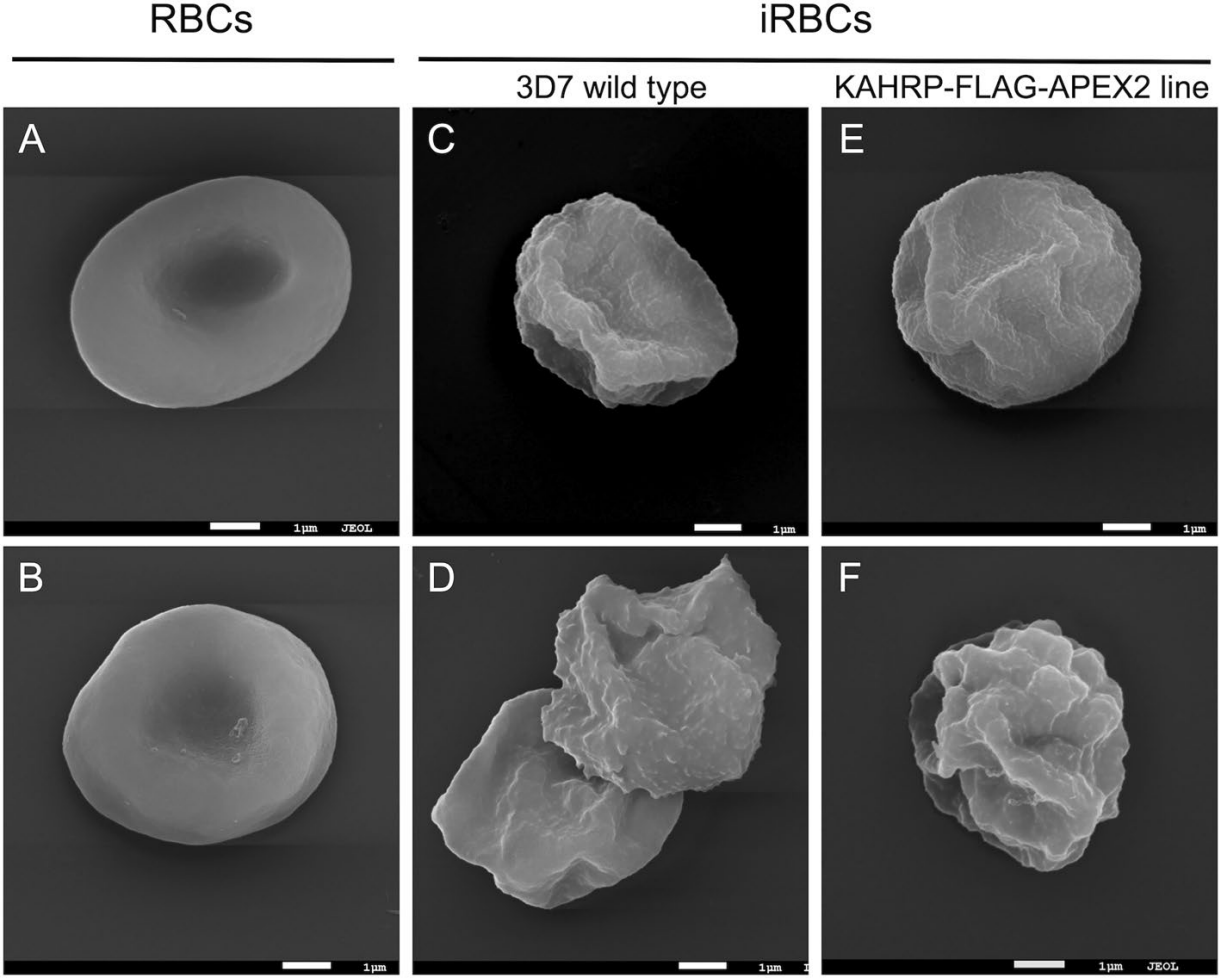
Scanning electron microscopy of *P. falciparum*-infected erythrocytes displaying the knob morphology. Uninfected RBCs (**A**, **B**) and Percoll-purified trophozoite/schizont iRBCs of 3D7 (**C**, **D**) and KAHRP-FLAG-APEX2 (**E**, **F**) lines. Scale bars: (**A**–**F**) 1 µm.

To confirm that the addition of the FLAG-APEX2 tags did not affect KAHRP function, the presence of knobs in the trophozoite/schizont-iRBCs of the transfectant line was examined by scanning electron microscopy (SEM) compared to the 3D7 parental strain and uninfected RBCs. SEM showed a typical knobbed surface on both 3D7 and transfectant trophozoite/schizont-iRBCs (Fig. 4). This pattern was consistent with previous studies^15,18,31^, indicating that KAHRP-FLAG-APEX2 is correctly expressed and can support the production of knob structures in the iRBC membrane. To test for any impact on cytoadherence, we incubated human umbilical vein endothelial cells (hUVECs) with both 3D7 and transfectant trophozoite/schizont-iRBCs, and saw adherence of iRBCs from both strains, whereas no adherence was seen when hUVECs were exposed uninfected RBCs (Fig. S3A–C)^40^. There was no statistical difference in the binding frequency of iRBCs from 3D7 and KAHRP-FLAG-APEX2 transfectant lines to hUVECs (Fig. S3D). Together with the SEM data, this suggests addition of the FLAG-APEX2 tags to the endogenous KAHRP protein has no discernible impact on knob production or function in cytoadherence.

### Proteomic identification of proteins biotinylated by KAHRP-FLAG-APEX2

Biotinylated proteins were enriched using streptavidin magnetic beads from triplicate samples from KAHRP-FLAG-APEX2 line, with material purified in parallel from both the BP+/H_2_O_2_+ condition and the BP−/H_2_O_2_+ condition to act as a negative control. Proteins were eluted from the beads, precipitated, alkylated and digested with trypsin as described in the Methods, and subjected to LC–MS/MS using an Orbitrap mass analyser. Proteins were identified by searching peptide sequences against the 3D7 proteome, with *H. sapiens* erythrocyte databank.

40,327 peptides were identified in the KAHRP-FLAG-APEX2 BP+/H_2_O_2_+ condition and 35,845 peptides were identified in the KAHRP-FLAG-APEX2 BP−/H_2_O_2_+ condition (Table S1). The high number of peptides identified in the pull-downs from both conditions does not appear to correlate with the Western blotting (Fig. 2A) and IFA results (Fig. 3B), which both indicate that biotinylated proteins are present at much higher abundance in the BP+/H_2_O_2_+ condition than the BP−/H_2_O_2_+ condition. However, this is likely due to non-specific purification of non-biotinylated proteins during enrichment. Although the affinity between streptavidin and biotin substrate is strong^41,42^, theoretically favouring highly specific enrichment, nonspecific binding of non-biotinylated proteins to streptavidin beads is a known concern, mostly because of the amount and composition of the streptavidin coated beads used for the protocol^43^.

Given these potential sources of background caused by non-biotinylated proteins being enriched along with biotinylated proteins, we took a deliberately conservative and stringent approach to minimise the risk of false positives. We only considered proteins as potential KAHRP-FLAG-APEX2 interactors if we could detect that protein from at least 2 different peptides, where at least one must be a unique proteotypic peptide^44^ that contains a variable biotin-phenol (+ 361.14601) modification on Tyr residues, confirming that it was definitively biotinylated. We considered only unique biotinylated peptides by which we could assess the signature of the biotin-phenol fragmented ions on the mass spectra as already reported elsewhere^42^. This analysis identified a total of 42 non-redundant biotinylated peptides that were found exclusively in the KAHRP-FLAG-APEX2 BP+/H_2_O_2_+ condition. These peptides corresponded to 8 *H. sapiens* erythrocyte proteins and 22 *P. falciparum* proteins, including KAHRP-FLAG-APEX2 (Table S2).

Remarkably, many proteins observed localizing at MC by IFA or proteomic analyses in previous studies were found biotinylated in our dataset. Among these proteins are ring exported protein-1 (REX1)^45^, ring exported protein-2 (REX2)^46^, skeleton-binding protein 1 (SBP1)^47^, early transcribed membrane protein 10.2 (ETRAMP10.2)^48^, small exported membrane protein (SEMP1)^49^ and parasite-infected erythrocyte surface protein (PIESP2)^48^. It is also noteworthy to mention that host proteins were also tagged by KAHRP-FLAG-APEX2. Among these proteins are α/β-spectrin, Protein 4.1, glycophorin C and band 3. These proteins are intimately associated with RBC cytoskeleton, which could explain their labelling considering KAHRP as main knob constituent^17^ (Table 1). Once KAHRP is also transported through MC^47^, it is not at all surprising that other MC proteins may interact with KAHRP at some time point during their export into iRBC membrane. Thus, this study remarks the first report of such direct interactions during in vitro schizogony in *P. falciparum* development.

**Table 1:** List of the candidate neighbouring parasite protein partners of the knob-associated histidine rich protein during schizogony in *P. falciparum.*

PlasmoDB accession^1^	Description	Exported? (PlasmoDB)^2^	PEXEL?^3^	PEXEL Match Location^3^	PEXEL Match Sequence^3^	SignalP^4^	SecretomeP^5^	TMHMM^6^	Phobius^7^
PF3D7_1444300	1-acyl-sn-glycerol-3-phosphate acyltransferase, putative	–	–	–	–	–	–	Yes	Yes
PF3D7_0202000	Knob-associated histidine-rich protein*	Yes	Yes	(54–58)	RTLAQ	–	Yes	–	Yes
PF3D7_1033200	Early transcribed membrane protein 10.2	–	–	–	–	Yes	–	Yes	Yes
PF3D7_1002100	EMP1-trafficking protein	–	Yes	(57–61)	RLLSE	–	Yes	Yes	Yes
PF3D7_0730900	EMP1-trafficking protein	–	Yes	(87–91)	RSLTE	–	Yes	Yes	Yes
PF3D7_1016300	GBP130 protein	Yes	Yes	(84–88)	RILAE	–	Yes	Yes	Yes
PF3D7_1105000	Histone H4	–	–	–	–	–	–	–	–
PF3D7_0500800	Mature parasite-infected erythrocyte surface antigen	–	Yes	(75–79)	RILSE	–	–	–	Yes
PF3D7_1335100	Merozoite surface protein 7	–	–	–	–	Yes	–	–	–
PF3D7_1228600	Merozoite surface protein 9	–	–	–	–	Yes	–	–	–
PF3D7_1412100	Mini-chromosome maintenance complex-binding protein, putative	–	–	–	–	–	Yes	–	–
PF3D7_0501200	Parasite-infected erythrocyte surface protein	–	Yes	(43–47)	RTLAD	Yes	–	Yes	Yes
PF3D7_1408100	Plasmepsin III	–	–	–	–	–	–	Yes	Yes
PF3D7_0402000	Plasmodium exported protein (PHISTa), unknown function	Yes	Yes	(69–73)	RNLSE	–	Yes	Yes	Yes
PF3D7_1353100	Plasmodium exported protein, unknown function	Yes	Yes	(86–90)	RILTQ	–	Yes	Yes	Yes
PF3D7_0702500	Plasmodium exported protein, unknown function	–	–	–	–	–	Yes	Yes	Yes
PF3D7_1252100	Rhoptry neck protein 3	–	–	–	–	Yes	–	Yes	Yes
PF3D7_0935900	Ring-exported protein 1	–	–	–	–	–	Yes	Yes	Yes
PF3D7_0936000	Ring-exported protein 2	–	–	–	–	–	Yes	Yes	Yes
PF3D7_0207600	Serine repeat antigen 5	–	–	–	–	Yes	–	–	–
PF3D7_0501300	Skeleton-binding protein 1	–	–	–	–	–	Yes	Yes	Yes
PF3D7_0702400	Small exported membrane protein 1	–	–	–	–	–	Yes	Yes	Yes

*This protein ID refers to the chimeric sequence of KAHRP-FLAG-APEX2.

^1^Gene ID obtained from PlasmoDB database (https://plasmodb.org/plasmo/app/).

^2^Prediction performed in the web tool “identify genes based on exported protein” from PlasmoDB (https://plasmodb.org/plasmo/app/search/transcript/GenesByExportPrediction).

^3^Prediction performed in the web tool “identify genes based on protein motif pattern” from PlasmoDB (https://plasmodb.org/plasmo/app/search/transcript/GenesByMotifSearch). The search was made by using PEXEL motif (RxLx[E/Q/D]) previously established elsewhere^12,52,55^.

^4^Prediction performed in the SignalP algorithm (https://services.healthtech.dtu.dk/services/SignalP-5.0/).

^5^Prediction performed in the SecretomeP algorithm (https://services.healthtech.dtu.dk/services/SecretomeP-2.0/).

^6^Prediction performed in the TMHMM algorithm (https://services.healthtech.dtu.dk/services/TMHMM-2.0/).

^7^Prediction performed in the Phobius algorithm (https://phobius.sbc.su.se/).

### Bionformatic analysis of potential KAHRP-interacting proteins

KAHRP is exported through the *Plasmodium* Translocon of EXported proteins (PTEX) localized at parasitophorous vacuole membrane (PVM) during its transport into the iRBCs^12,50,51^. Many *P. falciparum* proteins exported by this mechanism possess a conserved motif (RxLxE/Q/D), frequently referred as the *Plasmodium* EXport ELement (PEXEL) motif, at their N-terminal end^11,12,52^, and 8 of the 22 biotinylated proteins from our dataset were predicted to have a PEXEL motif at their N-terminal end (Table 1). Among these proteins are PTP4 and 5, *Pf*EMP1-trafficking proteins 4 and 5 (PF3D7_0730900 and PF3D7_1002100), GBP130 (PF3D7_1016300), MESA (PF3D7_0500800), PIESP2 (PF3D7_0501200) and KAHRP-FLAG-APEX2 (named as its endogenous gene name, knob-associated histidine rich protein, PF3D7_0202000).

Overall, a total of 6 and 12 of the 22 potential parasite KAHRP-interacting proteins were predicted to be secreted by classical or non-classical pathways by SignalP and SecretomeP, respectively. Moreover, 15 and 16 of the 22 potential parasite KAHRP-interacting proteins were predicted to contain at least one transmembrane protein domain by TMHMM and Phobius, respectively (Table S3). Altogether, 21 proteins are predicted to have an extracellular or cell surface localization corresponding to 95% of the 22 potential parasite KAHRP interactors identified. Only protein histone H4 (PF3D7_1105000) was not predicted to be exported by any algorithm used (Table 1).

**Figure 5:**
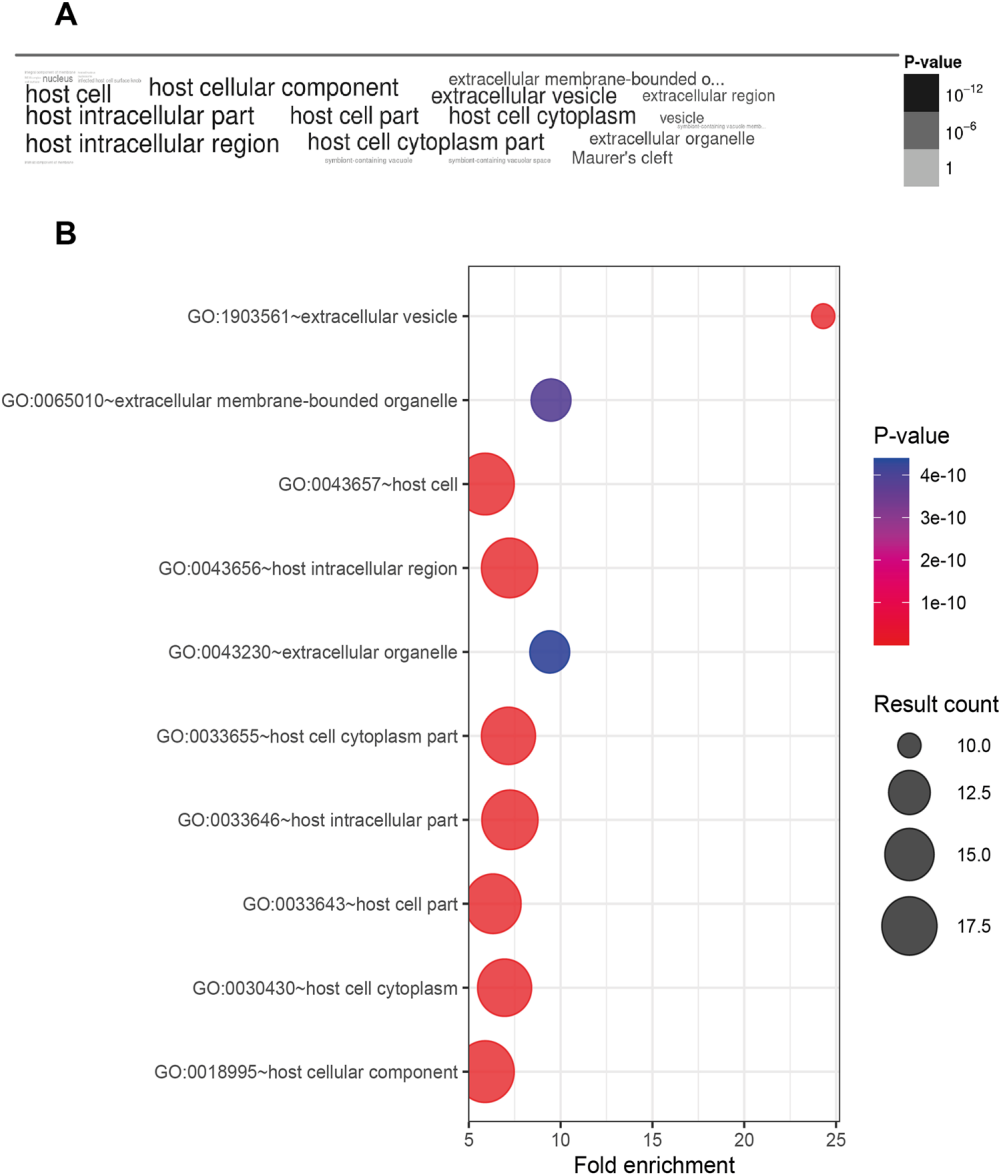
GO term annotation. (**A**) GO Cloud obtained from PlasmoDB (https://plasmodb.org/plasmo/app/) corresponding to the “Cellular Component” terms annotated. (**B**) Bubble plot from top 10 GO terms with most significant p-values from panel (**A**).

These 22 potential parasite KAHRP-interacting proteins were also GO-categorized in PlasmoDB (https://plasmodb.org/plasmo/app) to assess the “Cellular Compartment” ontology terms. A total of 24 GO terms for “Cellular Compartment” were returned from our data (Table S4). A significant enrichment was found in the expected GO terms. The most overrepresented GO terms statistically significant in the WordCloud and Bubble plot were related to the host environment (Fig. 5). Together, these bioinformatic approaches establish that the majority of the parasite proteins identified as candidate KAHRP interactors were secreted, exported and/or associated with membranes.

## Discussion

In this work, we have used APEX2-based proximity biotin-labelling and shotgun proteomics to identify proteins that are candidate interactors of *P. falciparum* KAHRP, the critical component of the knobs that form in the iRBC membrane and play a key role in cytoadherence. The APEX2 promiscuous biotinylation domain was integrated into the *P. falciparum* genome in-frame with the endogenous KAHRP protein, and electron microscopy and cytoadherence assays both detected no effect of the FLAG-APEX2 tag on either knob formation or function (Fig. 4 and Fig. S3), suggesting that KAHRP remained fully functional. After labelling assays, Western blotting confirmed that a set of proteins were biotinylated in this line only when the substrate BP was added, followed by H_2_O_2_ to allow oxidation (Fig. 2A). IFA confirmed that these biotinylated proteins were predominantly found in subcellular structures within the iRBC (Fig. 3C). By contrast, in the FLAG-APEX2 line with episomally expressed APEX2 that is not fused to any endogenous protein and hence is cytosolic, the APEX2 was active (Fig. S1) but the localisation of biotinylated proteins were intracellular (Fig. S2). No labelling was also detected in a wildtype control line (Fig. S1). Based on these data, APEX2 has significant promise to identify interacting proteins in *P. falciparum.*

Despite these clear results from Western blotting and IFA, enrichment of biotinylated proteins by streptavidin beads followed by identification using mass spectrometry revealed high levels of non-specific background in both conditions of the KAHRP-FLAG-APEX2 transfectant. It was therefore expected that there would be some level of non-specific background detectable by mass spectrometry and not by Western blotting/IFA, but there is no question that the level of background in the mass spectrometry data was a surprise. There is a consensus that the use of more streptavidin beads than necessary can increase the background by providing more surface area for nonspecific binders. Elution is also a bottleneck, specifically the difficulty of detaching biotinylated proteins from streptavidin, given the interaction is exceedingly strong^42,43,53^.

Given this significant issue with background, we applied highly stringent filters, assigning proteins as potential KAHRP interactors only if they were identified by two peptides, with at least one being unique containing a biotinylated Tyr, in at least one of the test replicates, and in none of the control replicates. From these unique biotinylated peptides, an additional criteria were employed: the presence of MS/MS spectral data of fragmentation ion assignment of dehydrobiotin (m/z 227.08), immonium of the bpY (m/z 497.22) and/or immonium of the bpY with loss of –NH_3_ (m/z 480.195), as already reported^42^.

This conservative and stringent approach identified with high confidence only 42 exclusive non-redundant biotinylated peptides from KAHRP-FLAG-APEX2 BP+/H_2_O_2_+ condition, representing a total of 22 parasite and 8 host proteins. It is important to be clear on the limitations and outlines of this dataset. Some interacting proteins may have biotinylation sites that are inaccessible, either because their coiled three-dimensional structure means that the tyrosine residues facing inwards, or they are membrane proteins with little of their sequence exposed to APEX2 activity. Moreover, some proteins that are more transient KAHRP partners but were not in the vicinity of KAHRP when the ‘snapshot’ of the APEX2-based biotinylation was carried out may be missing.

These caveats emphasise that there are undoubtedly false negatives, but there are multiple reasons to be confident that our dataset has captured true KAHRP interactors. To reach the host cell membrane, many parasite proteins exported across the parasitophorous vacuole membrane towards MC need to be processed by PTEX machinery^51,54^. Proteins exported by this mechanism contain a PEXEL motif at N-terminal end^12,55^. KAHRP possesses a PEXEL motif and is processed by this mechanism^12,52^, and transiently carried over into MC^47^. Among our data, beyond KAHRP, several other proteins identified as potential KAHRP interactors also have a PEXEL motif: PTP4 and 5, GBP130, MESA, PIESP2, PHISTa, and *Plasmodium* exported protein (PF3D7_1353100) (Table 1)^11,12^. Although we cannot establish whether these proteins were tagged by KAHRP-FLAG-APEX2 before, during or after translocation through PTEX machinery, the presence of proteins widely known to be exported through this machinery increases confidence in the hits identified in our dataset.

KAHRP-FLAG-APEX2 was confirmed by IFA to be localized in punctate structures (likely MC), as were biotinylated proteins catalysed by it (Fig. 3A). As mentioned already, several of the exported proteins we identified as putative KAHRP interactors are known to be found in the MC, such as REX1^45^, REX2^46^, SBP1^56^, ETRAMP10.2^48^, SEMP1^49^ and PIESP2^48^ (Table 1). Interestingly, all these aforementioned proteins are characterized as PEXEL-Negative Exported Proteins (PNEPs)^57^. Given KAHRP is a true PEXEL-exported protein^12,52^, it is possible that labelling of these PNEPs indicates that some export PEXEL proteins and PNEPs are shared after PEXEL proteins have exited from the PTEX machinery.

Some of the proteins identified in our dataset, such as REX1^45^, SBP1^56^ and; PTP4 and 5^11^, are already known to be involved in PfEMP1 trafficking. PfEMP1 is often associated with KAHRP, supporting knob formation and/or cytoadherence to the host endothelium^18,47^. Some reports have indicated that KAHRP and PfEMP1 could interact with each other^58,59^, but this statement has been controversial. Structurally, PfEMP1 is a transmembrane protein found at the MC periphery prior redistribution to the iRBC membrane^35^. Moreover, the intracellular portion ATS (acidic terminal segment) at C-termini from PfEMP1, supposedly responsible to create mechanical connections once in the iRBC membrane, does not bind to KAHRP^60^. These topological and biochemical features of PfEMP1 would be reasonable to explain its absence in our dataset, once KAHRP-FLAG-APEX2 would not biotinylate it.

Other structures have been implicated in parasite protein trafficking within the infected erythrocyte, such as transient J-dots/Maurer’s clefts (JAM) complex trafficking^61^. One potential KAHRP interactor identified in our data, the *Plasmodium* exported protein (PF3D7_0702500), has been identified as resident both in the MC and also in the JAM complex^61^. In addition, KAHRP has also been observed in proteomic analysis of extracellular vesicles (EVs) released from ring-stage iRBCs, as have other putative KAHRP interactors found in our dataset such as GBP130, histone H4 and MESA^62^.

Several host proteins were also confirmed to be biotinylated in our dataset possibly concentrated in assembled knobs (Table S1). For instance, KAHRP was already reported to interact with domains 10–14 of β-spectrin, proximal to the spectrin-ankyrin complex^16^, and N-5 fragment (repeat 4) of α-spectrin^63^. Both α and β-spectrin were found biotinylated in our dataset (Table S1). It is also known that spectrin interact with Protein 4.1^64^, and Protein 4.1 is also biotinylated by KAHRP-FLAG-APEX2 in our data set, suggesting a proximity tagging that includes the 4.1-spectrin-KAHRP network in knobs and adjoining erythrocyte cytoskeleton. In addition, other *P. falciparum* proteins that are biotinylated in our findings have previously been shown to interact with Protein 4.1, specifically *Plasmodium* exported protein (PHISTa, PF3D7_0402000)^65^ and mature parasite infected erythrocyte surface antigen (MESA)^66,67^. Two additional erythrocyte proteins, glycophorin C and band 3, which are known to form a direct or indirect bridge with Protein 4.1 and spectrin, respectively^68,69^, were also biotinylated by KAHRP-FLAG-APEX2. These findings confirm an intimate relation between KAHRP-FLAG-APEX2 and cytoskeleton proteins from host, corroborating KAHRP location and knob architecture in *P. falciparum*-infected erythrocytes.

**Figure 6:**
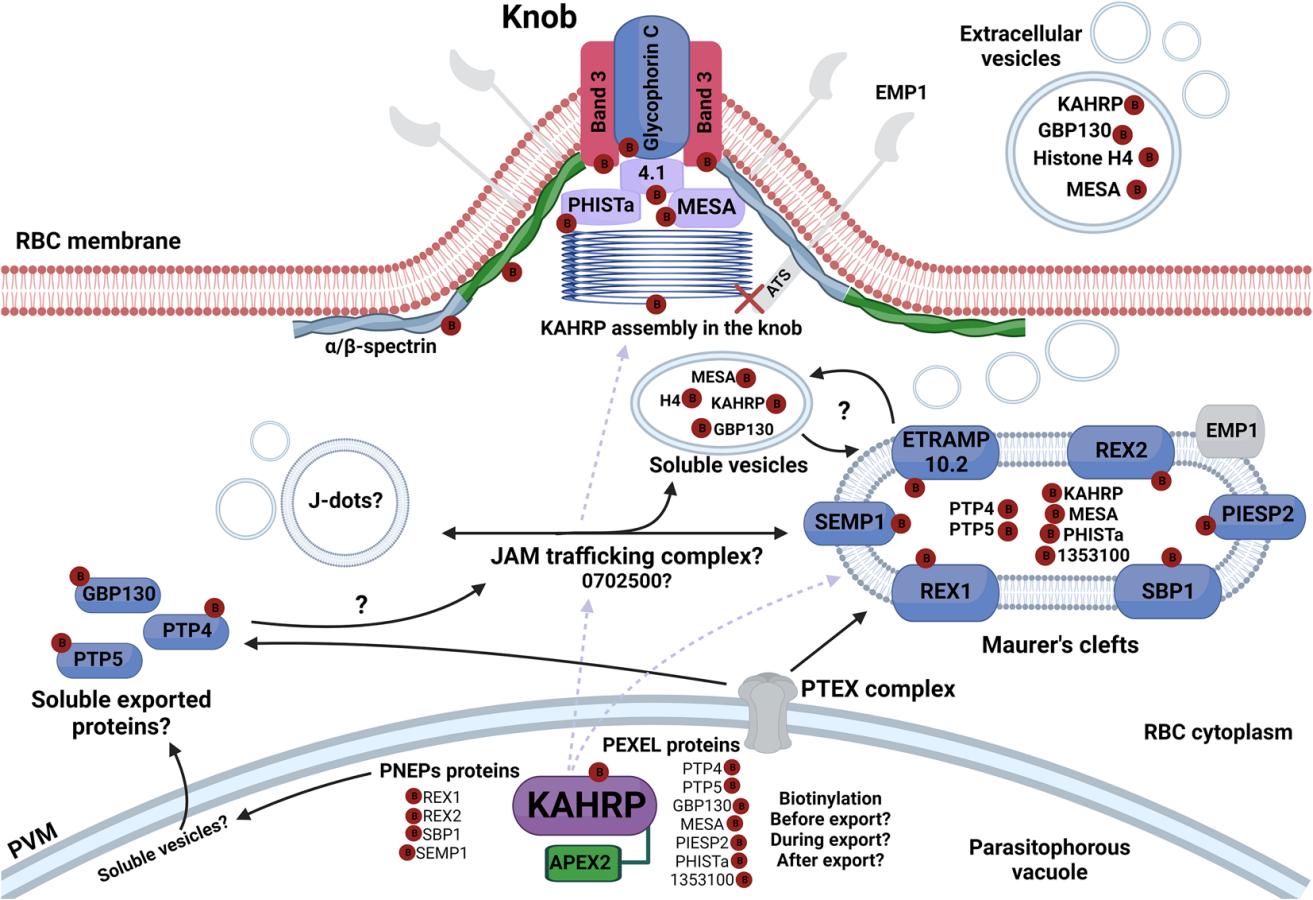
Diagram of potential cellular locations for interaction between KAHRP and parasite and host cell partners during protein export. Based on our proteomic data, APEX2-tagged KAHRP has biotinylated a set of proteins. Different trafficking routes throughout iRBC, including translocation via the PTEX complex and possibly alternative export pathways (e.g. PNEP proteins) are proposed to be used by KAHRP until its destination at knobs under the RBC membrane. Identified biotinylated proteins are labelled by a red circle with the letter B. PfEMP1, the main partner of KAHRP is described as a periphery protein found in the MC and its ATS domain does not interact with KAHRP. These topological features probably hampered APEX2 biotinylation. Other MC proteins, such as SBP1, PIESP2, REX1, REX2, SEMP1 and ETRAMP10.2 were found biotinylated in this study in agreement with the literature. Moreover, host proteins such as α/β-spectrin, protein 4.1, glycophorin and band 3 that compose cytoskeleton erythrocyte proteins were also biotinylated by KAHRP-APEX2, confirming the involvement of these proteins on the knob architecture. It is known that MC and J-dots comprise the JAM trafficking complex. One protein from our dataset found in this trafficking hub connecting these structures is the *Plasmodium* exported protein (PF3D7_0702500). Of special note, KAHRP and other proteins, such as GBP130, histone H4 and MESA, which were also found in our dataset as biotinylated, were reported to be in extracellular vesicles from ring-stage parasites, suggesting that protein export mechanisms in *P. falciparum* is much more complex than presumed.

In summary, while the biotin-based enrichment used clearly had issues of non-specific binding, our stringent protein identification approach established a list of high confidence list of potential KAHRP interacting proteins that include multiple known exported proteins, as well as host proteins that are known to be enriched in knobs. A schematic representation of how these proteins could interact in spatial and temporal context with KAHRP, either stably (for example by co-assembly into knobs) or transiently (by co-export using different cellular pathways) is proposed in Fig. 6.

## Concluding remarks

Protein export in malaria parasites is undoubtedly complex, and is still a field of intense exploration across *Plasmodium* species. Maurer’s Clefts have been widely studied and viewed as a primary structure involved in protein export^12,13,33–35,70^, but recent reports have shown that alternative mechanisms exist^61,62,71,72^. A better understanding of the protein composition of these pathways is needed, and APEX2 and BioID strategies to establish protein proximity represent technological breakthroughs that could help with this^19,28–30^. Tagging endogenous KAHRP with APEX2 resulted in the biotinylation of parasite proteins already described as exported into the RBC, suggesting that they are neighbouring KAHRP in a given time point. Among our dataset there are likely both direct and transient interactors with KAHRP, which may help to give a broader comprehension in protein export. The study also clearly identifies the methodological challenges concerning biotinylated protein/peptide elution in these proximity-tagging approaches^42,43,53,73,74^. Overall, our work has showed for the first time that APEX2 technology can be used for tagging a protein target in *P. falciparum* during the erythrocyte cycle and that while it has broad potential, rigorous controls and stringent analysis are essential to interpretation.

## Materials and methods

### *P. falciparum* culture conditions

*P. falciparum* strain 3D7 parasites were originally obtained from Malaria Research and Reference Reagent Resource Centre (available at: www.mr4.org) and were continuously maintained in O^+^ human erythrocytes as previously described^75^. All methods were carried out by relevant guidelines and regulations. The use of erythrocytes from human donors for parasite culture was approved by the Ethics Committee of NHS Cambridgeshire 4 Research (15/EE/0253) and all donors supplied written informed consent.

### Generation of transfection vectors

Vectors were designed using SeqBuilder software (DNASTAR), and a two-plasmid CRISPR-Cas9 system was used. The gRNA and Cas9 were expressed using pDC2-cam-Cas9-U6-sgRNA-yDHODH (yeast dihydroorotate dehydrogenase)^76,77^. To target specifically the *kahrp* gene, the first ranked gRNA target site localised after the stop codon was identified using CHOPCHOP (https://chopchop.cbu.uib.no) and confirmed by sgRNA Designer (http://www.broadinstitute.org/rnai/public/analysis-tools/sgrna-design). The backbone plasmid pCC1-*h*DHFR was used to build the targeted vector. FLAG-APEX2 sequence was amplified from pcDNA3APEX2-NES plasmid (Addgene plasmid # 49386)^78^. The hsp86 3’ UTR sequence was amplified from pINT plasmid^79^. The final assembled vector pCC1-FLAG-APEX2-HSP86-3’UTR was obtained in a single Gibson assembly reaction The Homology repair regions to target the insert to the 3’-end of *kahrp* were amplified from *P. falciparum* 3D7 genomic DNA, which was extracted and purified using a QIAamp DNA blood mini kit (QIAGEN). *Sac*I and *Afl*II digested homology region 1 (HR1) and *EcoR*I and *Nco*I digested homology region (HR2) were subcloned into multiple cloning site 1 and 2, respectively, of pCC1-FLAG-APEX2-HSP86-3’UTR. The resulting vector contained the drug-selectable marker h*dhfr* (human dihydrofolate reductase), which confers resistance to the antifolate drug WR99210^80^. All primers used for cloning procedures in this study are listed in the Table S5. Plasmids for *P. falciparum* transfection were obtained from transformed *E. coli* TOP10 cells containing either pCC1-FLAG-APEX2-HSP86-3’UTR-KAHRP-HR1-HR2 or pDC2-cam-Cas9-U6-sgRNA_*kahrp*_-yDHODH and purified using Maxiprep kit (QIAGEN).

### Transfection of parasites

For the transfection in *P. falciparum*, 50 µg of each plasmid was co-precipitated using 3 volumes (v/v) of ethanol and 10% (v/v) of the initial volume of DNA solution of 3 M sodium acetate pH 5.2. The DNA pellet was resuspended and mixed in 100 µL of buffer P3 (Lonza) with 4 µL ATP (625 mM). A total of 100 µL of ring stage-iRBCs with a parasitemia higher than 10% were pelleted and washed in cold sterile cytomix^81^. The washed iRBCs were resuspended in the cytomix containing both DNA plasmids and electroporated with Lonza Nucleofector 4D using programme P3/CM-150^82^. After 3 weeks of drug pressure using 1.25 nM of WR99210 (Jacobus Pharmaceuticals Company, Inc.) for pCC1 selection and 1.5 µM of DSM-1 for pDC2 selection^82^, the first parasites were seen through blood smears. DNA extraction was performed using QIAamp DNA Blood Mini Kit (QIAGEN), and the cassette integration into the specific loci of the genomic DNA were assessed by PCR.

### Biotinylation assay

Biotinylation assays were carried out using both wildtype control strains, and the KAHRP-APEX2 tagged line. Parasites were routinely synchronized using sorbitol treatment. Before biotinylation around 2 mL of RBCs at 5% parasitemia (corresponding to 1 × 10^9^ iRBCs) of predominantly late stages (trophozoites/schizonts) were enriched by centrifugation over 63% (v/v) isotonic Percoll cushion (Percoll Plus 17-5445-01, GE Healthcare), with the interface, which contains primarily late-stage parasites, isolated. This parasite fraction was then divided in equal volumes between the different test conditions in order to have the same cell number in all conditions. The enriched parasites were resuspended in complete RPMI medium without phenol red, supplemented with or without 250 µM biotin-phenol (Iris Biotech, CDX-B0270), and incubated for 30 min at 37 ºC, 5% CO_2_ to allow biotinylation to occur. After the incubation period each condition was split in two, with half having 1 mM H_2_O_2_ added (final concentration; GE Healthcare) for 1 min, and half not. Samples from a total of four conditions were therefore generated—in the presence or absence of biotin-phenol (BP), and each one with or without H_2_O_2_. Immediately after H_2_O_2_ addition the reactions were quenched with an antioxidant solution containing 10 mM sodium azide (Sigma-Aldrich), 10 mM sodium ascorbate (Sigma-Aldrich) and 5 mM of Trolox (Sigma-Aldrich) in PBS. A fraction of the iRBCs were pelleted to make blood smears used for immunofluorescence assays. The remaining iRBCs were lysed with 0.1% (w/v) saponin (Sigma-Aldrich) in PBS containing EDTA-free protease inhibitor cocktail (cOmplete™, Roche), and the pellets (which contain both intact parasite and membrane material from the iRBC including Maurer’s Clefts) were frozen for subsequent use in Western blots and proteomics. Material from all four conditions were used in Western Blots, but only two were then used for subsequent pull-down of biotinylated proteins and proteomic analysis: BP+/H_2_O_2_+ and BP−/H_2_O_2_+.

### Protein extraction and pull-down of biotinylated proteins

For Western blotting and proteomic analyses, frozen saponin parasite pellets were thawed and then lysed by using 4–5 volumes of RIPA buffer containing 50 mM Tris–HCl pH 7.0 (Invitrogen™), 150 mM NaCl (Sigma-Aldrich), 0.1% (w/v) SDS (Invitrogen™), 0.5% (w/v) sodium deoxycholate (Sigma-Aldrich), 1% (v/v) Triton X-100 (Sigma-Aldrich), EDTA-free protease inhibitor cocktail (cOmplete™, Roche), 10 mM sodium azide (Sigma-Aldrich), 10 mM sodium ascorbate (Sigma-Aldrich) and 5 mM of Trolox (Sigma-Aldrich) in PBS^28–30^. The lysates were incubated for 30 min, 4 ºC, followed by a centrifugation at 15,000 × *g*, for 20 min, 4 ºC to remove insoluble material^26^. Biotinylated proteins were enriched from the soluble fraction using Pierce™ streptavidin magnetic beads (Thermo Scientific). Fifty microliters of slurry beads were added per sample and incubated for1 hour with mild agitation at room temperature. The beads were separated from the flowthrough by using MagJET separation rack (Thermo Scientific) and washed as follow: (1) 2 × 1.4 mL with RIPA buffer; (2) 1 × 1.4 mL with RIPA buffer with 1 M of urea and (3) 2 × 1.4 mL with RIPA buffer. Biotinylated proteins were eluted from the beads using 100 µL of a solution containing 20 mM DTT (GE Healthcare), 2 mM of biotin (Sigma-Aldrich) and NuPAGE™ LDS Sample Buffer supplemented with NuPAGE Sample Reducing Agent and NuPAGE™ Antioxidant (Invitrogen™), heated at 95 ºC, for 5 min^28–30^.

### NuPAGE™, Western-blot and streptavidin-blot

NuPAGE™ Novex™ 4–12% Bis–Tris protein gels in MES buffer (Invitrogen™) were used to separate 15–20 µg of RIPA soluble proteins, before blotting onto nitrocellulose membrane (GE Healthcare). After transfer, the membranes were stained with 0.1% (w/v) Ponceau S (Sigma-Aldrich) in 5% (v/v) of acetic acid (Sigma-Aldrich). After blocking with 5% (w/v) skimmed milk, nitrocellulose membranes were incubated with anti-FLAG coupled to horseradish peroxidase (HRP; Sigma-Aldrich; 1:800 dilution) or with streptavidin-HRP (Invitrogen™; 1:10,000 dilution). Blots were developed with Amersham ECL Prime chemiluminescence substrate (GE Healthcare) and fluorograms were collected.

### Immunofluorescence assays

Smears from KAHRP-APEX2 or WT parasites enriched for late stages were prepared on glass slides and dried for at least 30 min before fixing with 4% (v/v) paraformaldehyde (Sigma-Aldrich) for 20 min and washing once with PBS. Cells were permeabilized with 0.1% (v/v) Triton X-100 (Sigma-Aldrich) in PBS and blocked for 3 h at 8 °C using 3% (w/v) BSA in PBS. To identify the location of the KAHRP-APEX2 fusion, the parasites were probed with mouse monoclonal anti-FLAG M2 antibody (Sigma-Aldrich, F3165; 1:800 dilution) for 1 h at room temperature in blocking solution followed by staining with an Alexa Fluor 555-conjugated anti-mouse IgG goat antibody (Invitrogen™, A21424; 1:800 dilution). To identify proteins biotinylated following the biotinylation assay, slides were co-probed with Alexa-Fluor 488-conjugated streptavidin (Invitrogen™, S11223; 1:800 dilution) and IgG rabbit antibodies directly raised against *Pf*MAHRP or *Pf*ERD2 (MRA; 1:200 dilution for both) for 1 h at room temperature in blocking solution. After three washes in PBS, cells were incubated with Alexa-Fluor 546-conjugated anti-rabbit IgG donkey antibodies (Invitrogen™, A10040; 1:800 dilution). After a final wash, the slides were mounted with anti-fade mounting medium with 4′,6-diamidino-2-phenylindole dihydrochloride (DAPI, Molecular Probes). Confocal images were acquired with a LSM510 laser scanning confocal microscope (Zeiss).

### Scanning electron microscopy (SEM)

Trofozoites and schizonts from both 3D7 and KAHRP-FLAG-APEX2 lines were enriched by using Percoll cushion 63% (v/v) (Percoll Plus 17–5445-01, GE Healthcare). Knob selectivity was not performed as used in previous studies^15,83^. Cells were then washed with PBS and fixed with 2.5% (v/v) glutaraldehyde in 0.1 M sodium cacodylate buffer for 1 h. After fixation, cells were conditioned in 0.1 M sodium cacodylate. Following the procedure, cells were fixed in contrast solution (0.1% (w/v) OsO_4_ in 0.1 M sodium cacodylate buffer) at room temperature. After fixation, cells were washed with distilled water. Coverslips were prepared with poly-l-lysine to attach trophozoites/schizonts-iRBCs, followed by acetone gradient dehydration (50%, 70%, 90% and 100%). Critical drying of samples was performed by using CPD 030 critical point dryer (BALZERS, USA). Dried samples were mounted on suitable sample holders using conductive adhesive and taken to the SCD 500 metalizer (Leica, Germany). All analyses were performed in the JSM-7001F (JEOL, Japan) SEM microscope, and the images were collected at an increase of 10.000 × with 15.0 kv.

### Cytoadherence assay of *P. falciparum*-iRBCs to the human endothelial cells

hUVEC cultures were maintained in DMEM-F12 (Gibco™) medium, supplemented with 10% (v/v) fetal bovine serum (FBS, Gibco™) and 1% (v/v) penicillin/streptomycin solution (Sigma-Aldrich), at 37 ºC in 5% CO_2_ atmosphere. hUVEC cells were cultured until a confluence of about 70–80% were achieved. Only trophozoites/schizonts *P. falciparum*-iRBCs were used, obtained by Percoll enrichment as already described. A total of 5 × 10^3^ of hUVEC cells and ~ 50% parasitaemia of *P. falciparum* trophozoites/schizonts (adjusted for 0.5% (v/v) haematocrit) were used for cytoadherence assay. Briefly, hUVEC cells were seeded into 8-well Lab-Tek CC2 chamber slides (Nalge Nunc International) coated with 1% (w/v) gelatin (Sigma-Aldrich) during overnight. hUVEC cells were washed once with binding medium (RPMI medium supplemented with 0.5% (w/v) AlbuMAX II (Gibco™) and cell attachment was observed by PrimoVert invert microscope (Zeiss). Next, *P. falciparum* trophozoites/schizonts were seeded over hUVEC cells at 37ºC in 5% CO_2_ atmosphere, during 1 h. After incubation, cells were immediately washed four times with binding medium to remove unattached cells. hUVEC and *P. falciparum*-iRBCs were fixed with methanol and stained with InstantProv hematological panotic kit (NewProv) and observed in a 1000× oil immersion lens. The same protocol was performed by using uninfected red blood cells (uRBCs) as negative control. All assay was performed in four independent biological replicates. Parasite-like bodies were counted onto direct hUVEC membrane association^40^. For statistical comparisons, GraphPad Prism v. 5.04 was used for normality test, Mann–Whitney test and graphical representation. A comparison was statically significant when *p value* was ≤ 0.05.

### Proteomic analysis

After confirmation by streptavidin-HRP blot that the KAHRP-FLAG-APEX2 line was producing biotinylated proteins, independent biological triplicate samples from the conditions BP+/H_2_O_2_+ and BP−/H_2_O_2_+ were obtained, with the same independent triplicates samples from the same conditions also obtained from the wildtype 3D7 parasite strain as an additional control. All samples were enriched using streptavidin beads as described above, and eluted material precipitated with 4 volumes of cold acetone to the sample in a proportion of 80:20. The protein pellet was washed twice with cold ethanol 70% (v/v), followed with the air drying in the fume hood^84–86^. The protein pellet was then resuspended in 25 mM triethylammonium bicarbonate buffer (TEAB, Sigma-Aldrich), reduced with 5 mM dithiothreitol (GE Healthcare) for 30 min, at 55 ºC, followed by alkylation of disulfide bonds with 14 mM iodoacetamide (Sigma-Aldrich) for 40 min in the dark, at room temperature. Next, the sample was digested with trypsin gold (Promega) at 37 ºC, for 18 h, then desalted using a homemade desalting system of packed P-200 C18 columns (3 M™ Empore™) and Poros OligoR3 resin (Applied Biosystems™)^87^. The eluted peptides were dried in SpeedVac centrifuge (Eppendorf™) using LoBind® tubes (Eppendorf™) and stocked at -20 ºC until the day of usage.

Liquid chromatography (LC) was performed with an RSLCnano system (Ultimate 3000, Thermo Scientific) coupled online to an Orbitrap Exploris 480 mass spectrometer (Thermo Scientific). Peptides were trapped on a C18 column (75 μm inner diameter × 2 cm; nanoViper Acclaim PepMap™ 100, Thermo Scientific) with buffer A (2/98 MeCN/H_2_O in 0.1% formic acid) at a flow rate of 3.0 µL/min over 4 min. Separation was performed on a 50 cm × 75 μm C18 column (nanoViper Acclaim PepMap™ RSLC, 2 μm, 100 Å, Thermo Scientific) regulated to a temperature of 40 °C with a linear gradient of 3% to 29% buffer B (B: 100% MeCN in 0.1% formic acid, A: 100% H_2_O in 0.1% formic acid) at a flow rate of 300 nL/min over 91 min. MS full scans were performed in the ultrahigh-field Orbitrap mass analyzer in ranges m/z 375–1500 with a resolution of 120,000 at m/z 200. The top 20 most intense ions were subjected to Orbitrap for further fragmentation via high energy collision dissociation (HCD) activation and a resolution of 15,000 with the auto gain control (AGC) target set to 100%. We selected ions with charge state from 2+ to 6+ for screening. Normalized collision energy (NCE) was set at 30 and the dynamic exclusion of 40 s.

For identification, the data were searched against the *P. falciparum* strain 3D7 proteome databank (Proteome UP000001450, from August, 2021) with 5,456 entries downloaded from Uniprot (https://www.uniprot.org/). To this file, 438 entries that contained “red blood cells”, “hematopoiesis” and “erythrocytes” associated key words from the *Homo sapiens* proteome databank (Proteome UP000005640, from August, 2021) were manually added to identify potential erythrocyte proteins, and the KAHRP-FLAG-APEX2 chimeric sequence was also manually added. Enzyme specificity was set to trypsin and a maximum of two miss cleavages sites were allowed. Carbamidomethylation of cysteine (+ 57.02) was set as fixed modification. Oxidized methionine (+ 15.99), biotinyl-tyramide of tyrosine residues (+ 361.14601) and N-terminal acetylation (+ 42.01) were set as variable modifications^42^. Maximum allowed mass deviation was set to 10 ppm for monoisotopic precursor ions and 0.02 Da for MS/MS peaks. The resulting files were further processed using PEAKS Studio 7.0. FDR was set to 0.01 at the peptide level for the whole study. To determine a protein as “biotinylated”, a manual selection of 2 peptides, with at least one being unique, was used^44,88^. All identified proteins must have at least one unique peptide with a variable biotin-phenol (+ 361.14601) modification on Tyr residues in at least one of the test replicates. To ensure that all unique biotinylated peptides used to identify KAHRP candidate interactors are indeed tagged by biotinylation, a manual screening was performed to detect through MS/MS spectra data from fragmentation ions signature of dehydrobiotin (m/z 227.08), immonium bpY (m/z 497.22) and/or immonium bpY with loss of –NH_3_ (m/z 480.195), as described previously^42^. All intensities of these fragmentation ions were also listed.

### Bioinformatic analysis

Proteins predicted to contain the export PEXEL motif (RxLx[EQD]) were obtained from PlasmoDB (https://plasmodb.org/plasmo/app). Overrepresented Gene Ontology (GO) terms were predicted by GO Cloud tool through PlasmoDB (https://plasmodb.org/plasmo/app) databank. The bubble plots from those enriched GO terms were obtained from R programming language by using ggplot2 package^89^. The potential of identified proteins to be secreted and/or exported to the extracellular environment of all proteins were assessed using the SignalP^90,91^ and SecretomeP WebServer^92,93^. For transmembrane protein prediction, TMHMM^94^ and Phobius^95^ were additionally employed.

### Supplementary Information

Supplementary Information.

## Data Availability

The mass spectrometry proteomics data have been deposited to the ProteomeXchange Consortium via the PRIDE partner repository with the dataset identifier PXD047791^96^.

## References

[1] WHO. *World Malaria Report*. *World Health Organization* (2022).

[2] Partnership SCT (2015). Efficacy and safety of RTS, S/AS01 malaria vaccine with or without a booster dose in infants and children in Africa: Final results of a phase 3, individually randomised, controlled trial. The Lancet.

[3] Olotu A (2016). Seven-year efficacy of RTS, S/AS01 malaria vaccine among young African children. New Engl. J. Med..

[4] Datoo MS (2021). Efficacy of a low-dose candidate malaria vaccine, R21 in adjuvant Matrix-M, with seasonal administration to children in Burkina Faso: A randomised controlled trial. The Lancet.

[5] Ippolito MM, Moser KA, Kabuya J-BB, Cunningham C, Juliano JJ (2021). Antimalarial drug resistance and implications for the WHO global technical strategy. Curr. Epidemiol. Rep..

[6] Global Malaria Programme: WHO Global. *World Malaria Report*. *World Health Organization* (2020).

[7] Wassmer SC, Grau GER (2017). Severe malaria : What ’ s new on the pathogenesis front ?. Int. J. Parasitol..

[8] Spillman NJ, Beck JR, Goldberg DE (2015). Protein export into malaria parasite-infected erythrocytes: Mechanisms and functional consequences. Annu. Rev. Biochem..

[9] Moxon CA, Gibbins MP, McGuinness DDAM, Marti M (2020). New insights into malaria pathogenesis. Annu. Rev. Pathol.: Mech. Dis..

[10] Scherf A, Lopez-Rubio JJ, Riviere L (2008). Antigenic variation in *Plasmodium falciparum*. Annu. Rev. Microbiol..

[11] Maier AG (2008). Exported proteins required for virulence and rigidity of *Plasmodium falciparum*-infected human erythrocytes. Cell.

[12] Marti M (2004). Targeting malaria virulence and remodeling proteins to the host erythrocyte. Science.

[13] Lanzer M, Wickert H, Krohne G, Vincensini L, Braun-Breton C (2006). Maurer’s clefts: A novel multi-functional organelle in the cytoplasm of *Plasmodium falciparum*-infected erythrocytes. Int. J. Parasitol..

[14] Subramani R (2015). *Plasmodium falciparum*-infected erythrocyte knob density is linked to the PfEMP1 variant expressed. mBio.

[15] Looker O (2019). The knob protein KAHRP assembles into a ring-shaped structure that underpins virulence complex assembly. PLoS Pathog..

[16] Cutts EE (2017). Structural analysis of *P. falciparum* KAHRP and PfEMP1 complexes with host erythrocyte spectrin suggests a model for cytoadherent knob protrusions. PLoS Pathog..

[17] Sanchez CP (2021). KAHRP dynamically relocalizes to remodeled actin junctions and associates with knob spirals in *Plasmodium falciparum*-infected erythrocytes. Mol. Microbiol..

[18] Crabb BS (1997). Targeted gene disruption shows that knobs enable malaria-infected red cells to cytoadhere under physiological shear stress. Cell.

[19] Roux KJ, Kim DI, Raida M, Burke B (2012). A promiscuous biotin ligase fusion protein identifies proximal and interacting proteins in mammalian cells. J. Cell Biol..

[20] Kimmel J, Kehrer J, Frischknecht F, Spielmann T (2022). Proximity-dependent biotinylation approaches to study apicomplexan biology. Mol. Microbiol..

[21] Boucher MJ (2018). Integrative proteomics and bioinformatic prediction enable a high-confidence apicoplast proteome in malaria parasites. PLoS Biol..

[22] Khosh-Naucke M (2018). Identification of novel parasitophorous vacuole proteins in *P. falciparum* parasites using BioID. Int. J. Med. Microbiol..

[23] Birnbaum J (2020). A Kelch13-defined endocytosis pathway mediates artemisinin resistance in malaria parasites. Science.

[24] Schnider CB, Bausch-Fluck D, Brühlmann F, Heussler VT, Burda P-C (2018). BioID reveals novel proteins of the plasmodium parasitophorous vacuole membrane. mSphere.

[25] Chen AL (2015). Novel components of the Toxoplasma inner membrane complex revealed by BioID. mBio.

[26] Nadipuram SM, Thind AC, Rayatpisheh S, Wohlschlegel JA, Bradley PJ (2020). Proximity biotinylation reveals novel secreted dense granule proteins of *Toxoplasma gondii* bradyzoites. PLoS One.

[27] Geiger M (2020). Structural insights into PfARO and characterization of its interaction with PfAIP. J. Mol. Biol..

[28] Rhee H (2013). Proteomic mapping of mitochondria in living cells via spatially restricted enzymatic tagging. Science.

[29] Lee SY (2016). APEX fingerprinting reveals the subcellular localization of proteins of interest. Cell Rep..

[30] Lobingier BT (2017). An approach to spatiotemporally resolve protein interaction networks in living cells. Cell.

[31] Rug M, Prescott SW, Fernandez KM, Cooke BM, Cowman AF (2006). The role of KAHRP domains in knob formation and cytoadherence of *P. falciparum*—infected human erythrocytes. Blood.

[32] Diehl M (2021). Co-chaperone involvement in knob biogenesis implicates host-derived chaperones in malaria virulence. PLoS Pathog..

[33] Spycher C (2008). The Maurer’s cleft protein MAHRP1 is essential for trafficking of PfEMP1 to the surface of *Plasmodium falciparum*-infected erythrocytes. Mol. Microbiol..

[34] Spycher C (2003). MAHRP-1, a novel *Plasmodium falciparum* histidine-rich protein, binds ferriprotoporphyrin IX and localizes to the Maurer’s clefts. J. Biol. Chem..

[35] Spycher C (2006). Genesis of and trafficking to the Maurer’s Clefts of *Plasmodium falciparum*—infected erythrocytes. Mol. Cell Biol..

[36] Van Wye J (1996). Identification and localization of rab6, separation of rab6 from ERD2 and implications for an ‘unstacked’ Golgi, *Plasmodium falciparum*. Mol. Biochem. Parasitol..

[37] Krai P, Dalal S, Klemba M (2014). Evidence for a Golgi-to-endosome protein sorting pathway in *Plasmodium falciparum*. PLoS One.

[38] Hallée S, Boddey JA, Cowman AF, Richard D (2018). Evidence that the *Plasmodium falciparum* protein sortilin potentially acts as an escorter for the trafficking of the rhoptry-associated membrane antigen to the rhoptries. mSphere.

[39] Elmendorf HG, Haldar K (1993). Identification and localization of ERD2 in the malaria parasite *Plasmodium falciparum*: Separation from sites of sphingomyelin synthesis and implications for organization of the Golgi. EMBO J..

[40] Utter C, Serrano AE, Glod JW, Leibowitz MJ (2017). Association of *Plasmodium falciparum* with human endothelial cells in vitro. Yale J. Biol. Med..

[41] Chaiet L, Wolf FJ (1964). The properties of streptavidin, a biotin-binding protein produced by Streptomycetes. Arch. Biochem. Biophys..

[42] Kim DI (2018). BioSITe: A method for direct detection and quantitation of site-specific biotinylation. J. Proteome Res..

[43] Hung V (2016). Spatially resolved proteomic mapping in living cells with the engineered peroxidase APEX2. Nat. Protoc..

[44] Zhao Y, Lin YH (2010). Whole-cell protein identification using the concept of unique peptides. Genom. Proteom. Bioinf..

[45] McHugh E (2015). A repeat sequence domain of the ring-exported protein-1 of *Plasmodium falciparum* controls export machinery architecture and virulence protein trafficking. Mol. Microbiol..

[46] Haase S (2009). Sequence requirements for the export of the *Plasmodium falciparum* Maurer’s clefts protein REX2. Mol. Microbiol..

[47] Wickham ME (2001). Trafficking and assembly of the cytoadherence complex in *Plasmodium falciparum*-infected human erythrocytes. EMBO J..

[48] Vincensini L (2005). Proteomic analysis identifies novel proteins of the Maurer’s clefts, a secretory compartment delivering *Plasmodium falciparum* proteins to the surface of its host cell. Mol. Cell. Proteom..

[49] Dietz O (2014). Characterization of the small exported *Plasmodium falciparum* membrane protein SEMP1. PLoS One.

[50] Boddey JA, Moritz RL, Simpson RJ, Cowman AF (2009). Role of the Plasmodium export element in trafficking parasite proteins to the infected erythrocyte. Traffic.

[51] Koning-Ward TF (2009). A newly discovered protein export machine in malaria parasites. Nature.

[52] Boddey JA (2016). Export of malaria proteins requires co-translational processing of the PEXEL motif independent of phosphatidylinositol-3-phosphate binding. Nat. Commun..

[53] Schiapparelli LM (2014). Direct detection of biotinylated proteins by mass spectrometry. J. Proteome Res..

[54] Bullen HE (2012). Biosynthesis, localization, and macromolecular arrangement of the *Plasmodium falciparum* translocon of exported proteins (PTEX). J. Biol. Chem..

[55] Hiller NL (2004). A Host-targeting signal in virulence proteins reveals a secretome in malarial infection. Science.

[56] Maier AG (2007). Skeleton-binding protein 1 functions at the parasitophorous vacuole membrane to traffic PfEMP1 to the *Plasmodium falciparum*-infected erythrocyte surface. Blood.

[57] Heiber A (2013). Identification of new PNEPs indicates a substantial non-PEXEL exportome and underpins common features in *Plasmodium falciparum* protein export. PLoS Pathog..

[58] Waller KL, Cooke BM, Nunomura W, Mohandas N, Coppel RL (1999). Mapping the binding domains involved in the interaction between the *Plasmodium falciparum* knob-associated histidine-rich protein (KAHRP) and the cytoadherence ligand *P. falciparum* erythrocyte membrane protein 1 (PfEMP1). J. Biol. Chem..

[59] Ganguly AK, Ranjan P, Kumar A, Bhavesh NS (2015). Dynamic association of PfEMP1 and KAHRP in knobs mediates cytoadherence during Plasmodium invasion. Sci. Rep..

[60] Mayer C, Slater L, Erat MC, Konrat R, Vakonakis I (2012). Structural analysis of the *Plasmodium falciparum* erythrocyte membrane protein 1 (PfEMP1) intracellular domain reveals a conserved interaction epitope. J. Biol. Chem..

[61] Jonsdottir TK (2021). Characterisation of complexes formed by parasite proteins exported into the host cell compartment of *Plasmodium falciparum* infected red blood cells. Cell Microbiol..

[62] Sampaio NG (2018). Extracellular vesicles from early stage *Plasmodium falciparum*-infected red blood cells contain PfEMP1 and induce transcriptional changes in human monocytes. Cell Microbiol..

[63] Pei X (2005). Structural and functional studies of interaction between *Plasmodium falciparum* knob-associated histidine-rich protein (KAHRP) and erythrocyte spectrin. J. Biol. Chem..

[64] Schischmanoff PO (1995). Defining of the minimal domain of protein 4.1 involved in spectrin-actin binding. J. Biol. Chem..

[65] Parish LA, Mai DW, Jones ML, Kitson EL, Rayner JC (2013). A member of the *Plasmodium falciparum* PHIST family binds to the erythrocyte cytoskeleton component band 41. Malar. J..

[66] Kilili GK, LaCount DJ (2011). An erythrocyte cytoskeleton-binding motif in exported *Plasmodium falciparum* proteins. Eukaryot. Cell.

[67] Black CG (2008). In vivo studies support the role of trafficking and cytoskeletal-binding motifs in the interaction of MESA with the membrane skeleton of *Plasmodium falciparum*-infected red blood cells. Mol. Biochem. Parasitol..

[68] Marfatia SM, Lue RA, Branton D, Chishti AH (1994). In vitro binding studies suggest a membrane-associated complex between erythroid p55, protein 4.1, and glycophorin C. J. Biol. Chem..

[69] Chang SH, Low PS (2001). Regulation of the glycophorin C-protein 4.1 membrane-to-skeleton bridge and evaluation of its contribution to erythrocyte membrane stability. J. Biol. Chem..

[70] Maier AG, Cooke BM, Cowman AF, Tilley L (2009). Malaria parasite proteins that remodel the host erythrocyte. Nat. Rev. Microbiol..

[71] Batinovic S (2017). An exported protein-interacting complex involved in the trafficking of virulence determinants in Plasmodium-infected erythrocytes. Nat. Commun..

[72] Regev-Rudzki N (2013). Cell-cell communication between malaria-infected red blood cells via exosome-like vesicles. Cell.

[73] Berg-Luecke L, Gundry RL (2021). Assessment of streptavidin bead binding capacity to improve quality of streptavidin-based enrichment studies. J. Proteome Res..

[74] Renuse S (2020). Signature fragment ions of biotinylated peptides. J. Am. Soc. Mass Spectrom..

[75] Trager W, Jensen JB (1976). Human malaria parasites in continous culture. Science.

[76] Lim MYX (2016). UDP-galactose and acetyl-CoA transporters as Plasmodium multidrug resistance genes. Nat. Microbiol..

[77] Ganesan SM (2011). Yeast dihydroorotate dehydrogenase as a new selectable marker for *Plasmodium falciparum* transfection. Mol. Biochem. Parasitol..

[78] Lam SS (2014). Directed evolution of APEX2 for electron microscopy and proximity labeling. Nat. Methods.

[79] Nkrumah LJ (2006). Efficient site-specific integration in *Plasmodium falciparum* chromosomes mediated by mycobacteriophage Bxb1 integrase. Nat. Methods.

[80] Fidock DA, Wellems TE (1997). Transformation with human dihydrofolate reductase renders malaria parasites insensitive to WR99210 but does not affect the intrinsic activity of proguanil. Proc. Natl. Acad. Sci. U. S. A..

[81] Adjalley SH, Lee MCS, Fidock DA (2010). A method for rapid genetic integration into *Plasmodium falciparum* utilizing mycobacteriophage Bxb1 integrase. Methods Mol. Biol..

[82] Carrasquilla M (2020). Defining multiplicity of vector uptake in transfected Plasmodium parasites. Sci. Rep..

[83] Pasvol G, Wilson RJM, Brown J (1978). Separation of viable schizont-infected red cells of *Plasmodium falciparum* from human blood. Ann. Trop. Med. Parasitol..

[84] Jiang L, He L, Fountoulakis M (2004). Comparison of protein precipitation methods for sample preparation prior to proteomic analysis. J. Chromatogr. A.

[85] Fic E, Kedracka-Krok S, Jankowska U, Pirog A, Dziedzicka-Wasylewska M (2010). Comparison of protein precipitation methods for various rat brain structures prior to proteomic analysis. Electrophoresis.

[86] Puchades M, Westman A, Blennow K, Davidsson P (1999). Removal of sodium dodecyl sulfate from protein samples prior to matrix-assisted laser desorption/ionization mass spectrometry. Rapid Commun. Mass Spectrometry.

[87] Rappsilber J, Mann M, Ishihama Y (2007). Protocol for micro-purification, enrichment, pre-fractionation and storage of peptides for proteomics using StageTips. Nat. Protoc..

[88] Gupta N, Pevzner PA (2009). False discovery rates of protein identifications: A strike against the two-peptide rule. J. Proteome Res..

[89] Wickham H (2016). Ggplot2: Elegant Graphics for Data Analysis.

[90] Nielsen H, Tsirigos KD, Brunak S, von Heijne G (2019). A brief history of protein sorting prediction. Protein J..

[91] Almagro-Armenteros JJ (2019). SignalP 5.0 improves signal peptide predictions using deep neural networks. Nat. Biotechnol..

[92] Bendtsen JD, Kiemer L, Fausbøll A, Brunak S (2005). Non-classical protein secretion in bacteria. BMC Microbiol..

[93] Bendtsen JD, Jensen LJ, Blom N, Von Heijne G, Brunak S (2004). Feature-based prediction of non-classical and leaderless protein secretion. Protein Eng. Design Sel..

[94] Krogh A, Larsson B, Von Heijne G, Sonnhammer ELL (2001). Predicting transmembrane protein topology with a hidden Markov model: Application to complete genomes. J. Mol. Biol..

[95] Käll L, Krogh A, Sonnhammer ELL (2007). Advantages of combined transmembrane topology and signal peptide prediction-the Phobius web server. Nucleic Acids Res..

[96] Perez-Riverol Y (2022). The PRIDE database resources in 2022: A hub for mass spectrometry-based proteomics evidences. Nucleic Acids Res..

