# Supplementary material for: APEX2-based proximity proteomic analysis identifies candidate interactors for Plasmodium falciparum knob-associated histidine-rich protein in infected erythrocytes
Source: Sci Rep. 2024 May 16;14:11242. doi: 10.1038/s41598-024-61295-w (PMC11099048; doi:10.1038/s41598-024-61295-w)
Supplement: Supplementary file 1 — Supplementary Information. [file 41598_2024_61295_MOESM1_ESM.zip › SREP-23-04326-s3.docx]

**SUPPLEMENTARY DATA**

**Figure S1. Episomal vector construction and cytosolic APEX2 expression.** (A) Scheme of the plasmid pCC1-FLAG-APEX2 used as positive control for APEX2 expression. (B) Ponceau S stained nitrocellulose membrane (left panel), Western-blot (middle panel, anti-FLAG-HRP dilution at 1:800) and streptavidin-blot (right panel, streptavidin-HRP dilution at 1:10,000) of episome-based FLAG-APEX2 expressing line or wild-type *P. falciparum* 3D7 strain treated and/or not treated with BP and H_2_O_2_. In all cases material was generated by saponin lysis of iRBCs, which releases RBC cytosolic contents but retains parasite and exported membrane material, followed by solubilisation of all retained material using RIPA buffer. Legend for all gels/blots: first lane BP-/H_2_O_2_- (not treated with BP or H_2_O_2_); second lane BP-/H_2_O_2_+ (not treated with BP, treated with H_2_O_2_); third lane BP+/H_2_O_2_- (treated with BP but not treated with H_2_O_2_); fourth lane BP+/H_2_O_2_+ (treated with both BP and H_2_O_2_).

**Figure S2. IFA of the episome-based FLAG-APEX2 expressing line.** The images were obtained only from BP+/H_2_O_2_+ treated parasites. Both trophozoite (A) and schizont (B) stage parasites expressed APEX2 in the cytosol only, as expected given it is episomally expressed and not fused to any *P. falciparum* protein. Unlike in the KAHRP-FLAG-APEX2 line, no labelling corresponding to the biotinylated proteins was observed in the punctate structures nor in the periphery of infected erythrocytes.

**Figure S3. Cytoadherence assay.** No uninfected RBC (A), 3D7 wildtype (B) and KAHRP-FLAG-APEX2 transfectant (C) lines associated with hUVECs. Parasite-like bodies (black arrows) were counted per 100 hUVECs in four independent replicates (400 cells in total). Non-parametric tests were employed to assess the distribution of parasite-like bodies between two conditions. Therefore, Mann-Whitney test was chosen. Despite a similar patter of counting may be seen, no statiscal significance was observed (D). Scale bar: 10µm.

**Table S1. List of total peptides identified by MS from conditions:** (i) KAHRP-FLAG-APEX2 (BP+/H_2_O_2_+, both treated with BP and H_2_O_2_) and (ii) KAHRP-FLAG-APEX2 (BP-/H_2_O_2_+, not treated with BP and treated with H_2_O_2_).

**Table S2. Biotinylated proteins suggested as candidate protein-partner of KAHRP, based on their peptide exclusivity in the KAHRP-FLAG-APEX2 tagged line treated with BP+/H_2_O_2_+.**

**Table S3. List of biotinylated proteins with extracellular location predicted by *in silico* analysis by using SignalP, SecretomeP, TMHMM and Phobius.**

**Table S4. List of biotinylated proteins with annotated GO term prediction accordingly to PlasmoDB.**

**Table S5. List of primers used for cloning in this study for CRISPR-Cas9 vectors arrangement.**
