# Supplementary figures and images for: APEX2-based proximity proteomic analysis identifies candidate interactors for Plasmodium falciparum knob-associated histidine-rich protein in infected erythrocytes
Source: Sci Rep. 2024 May 16;14:11242. doi: 10.1038/s41598-024-61295-w (PMC11099048; doi:10.1038/s41598-024-61295-w)

**A**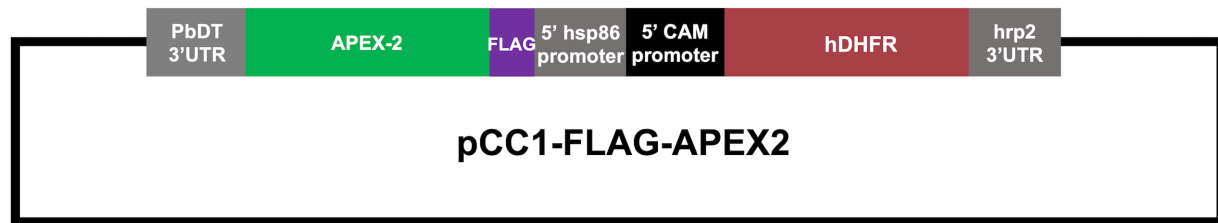**B**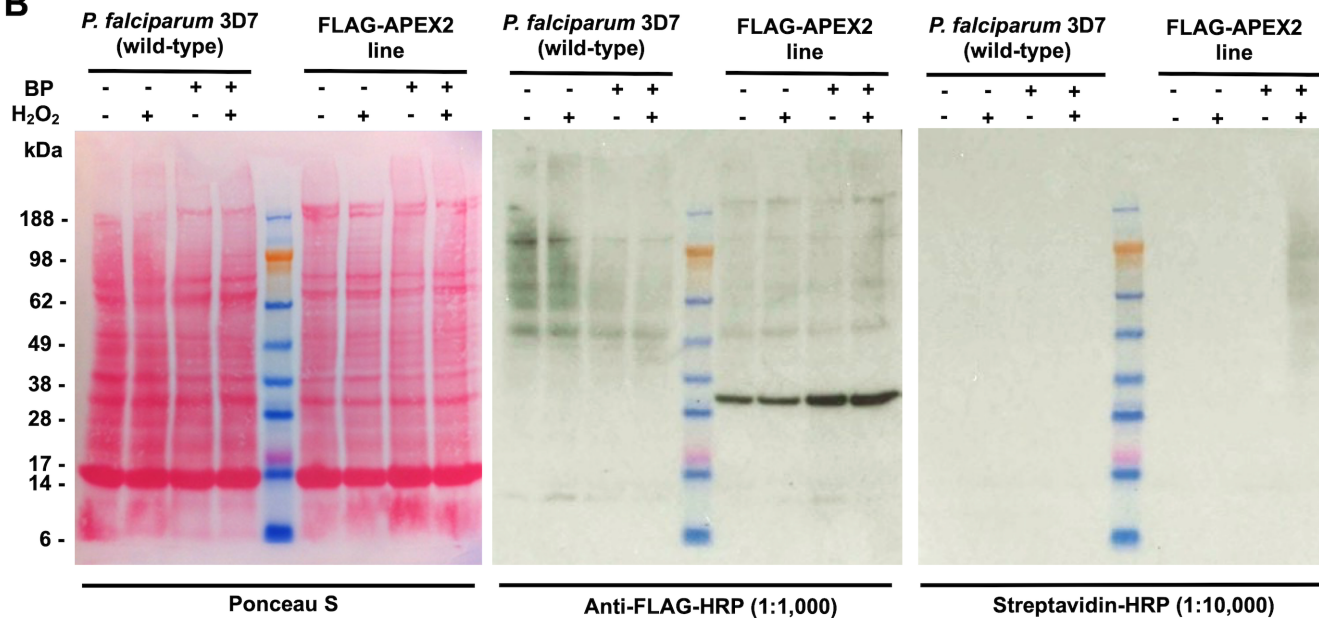**Figure S1**

Supplement: Supplementary file 1 — Supplementary Information. [file 41598_2024_61295_MOESM1_ESM.zip › SREP-23-04326-s0.pdf]

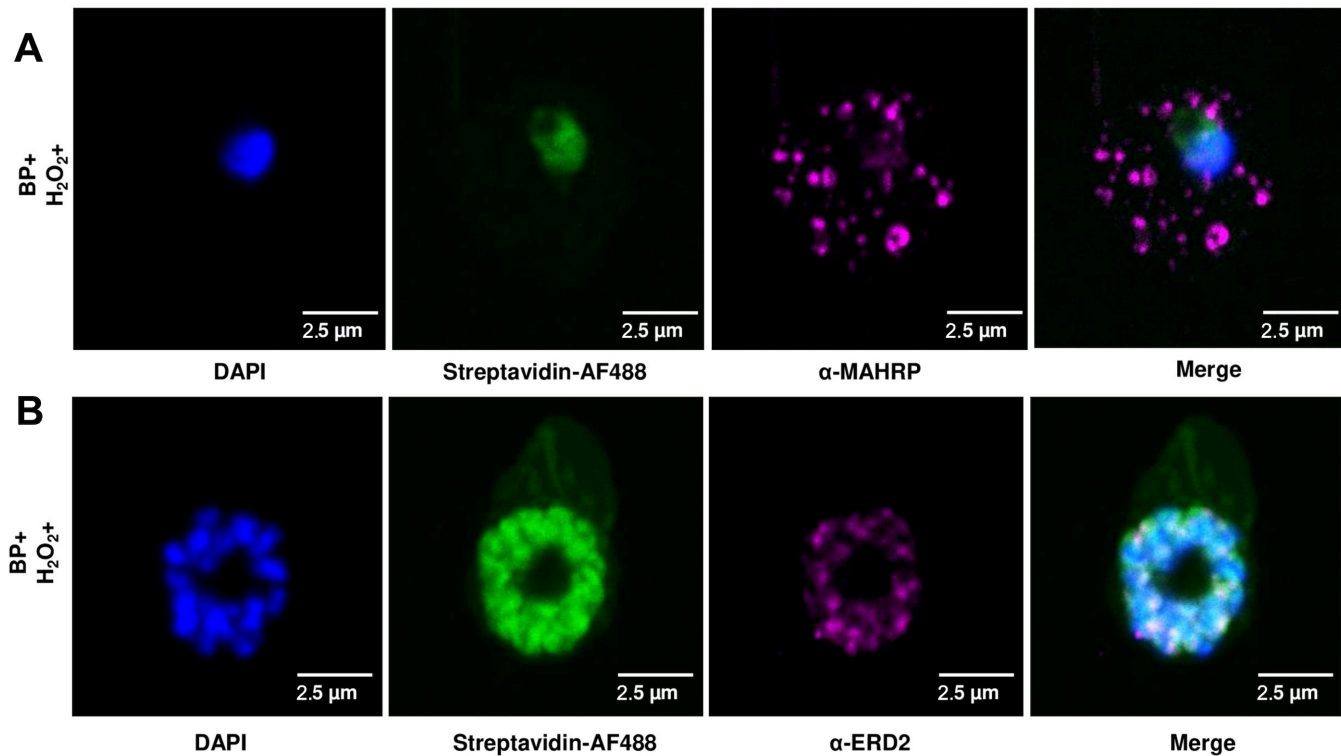

**Figure S2**

Supplement: Supplementary file 1 — Supplementary Information. [file 41598_2024_61295_MOESM1_ESM.zip › SREP-23-04326-s1.pdf]

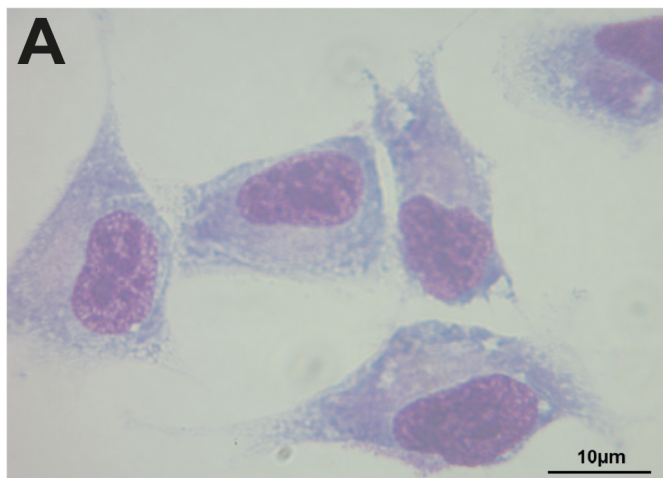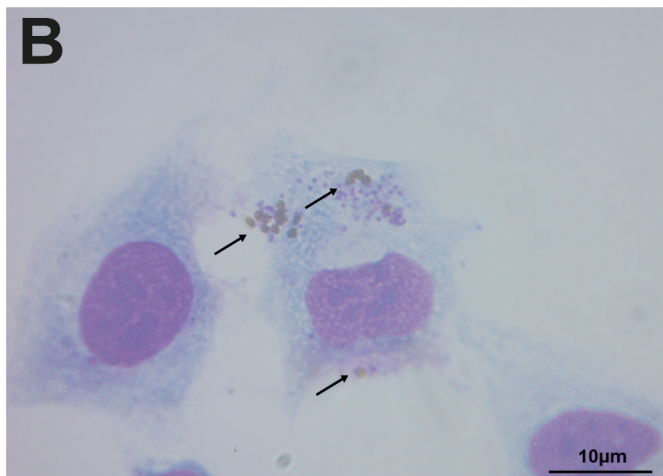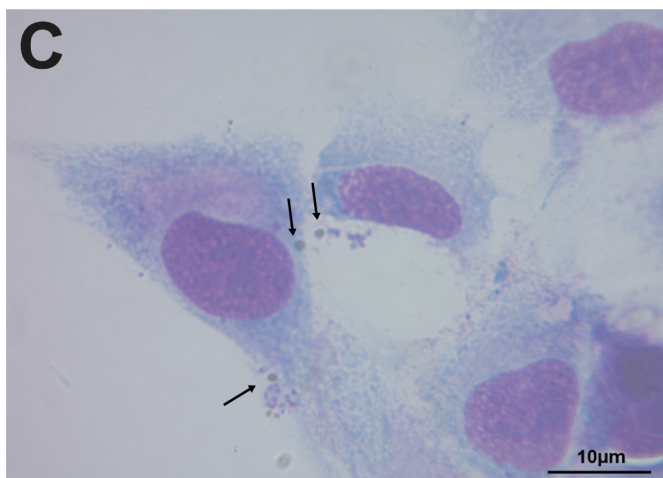

**D**

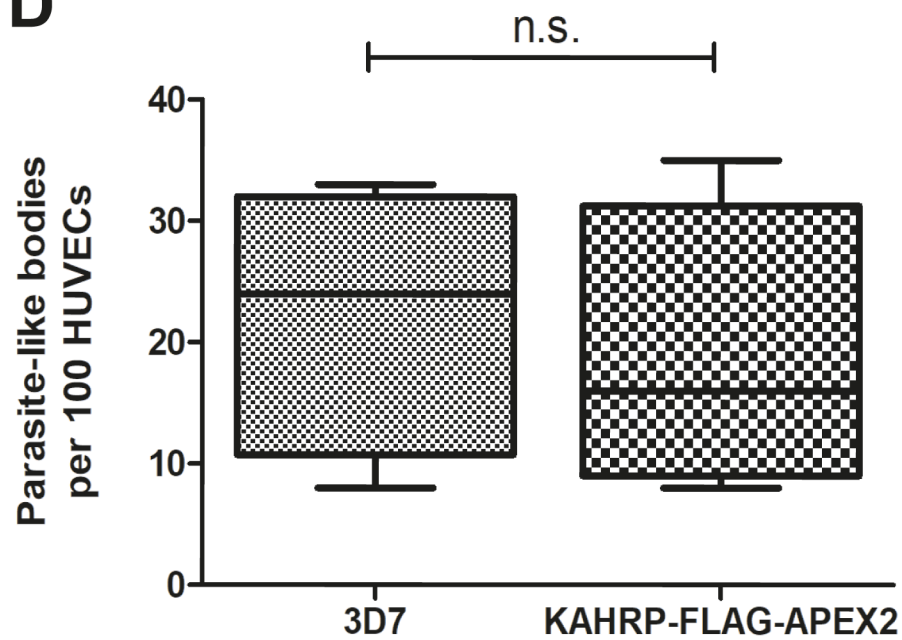

**Figure S3**

Supplement: Supplementary file 1 — Supplementary Information. [file 41598_2024_61295_MOESM1_ESM.zip › SREP-23-04326-s2.pdf]
